# Structural Analysis of Amylin and Amyloid β Peptide Signaling in Alzheimer’s Disease

**DOI:** 10.3390/biom15010089

**Published:** 2025-01-09

**Authors:** Longsheng Xie, Christopher Lockhart, Steven R. Bowers, Dmitri K. Klimov, Mohsin Saleet Jafri

**Affiliations:** 1 School of Systems Biology, George Mason University, Fairfax, VA 22030, USA; lxie2@gmu.edu (L.X.); clockha2@gmu.edu (C.L.);; 2 Center for Biomedical Engineering and Technology, University of Maryland School of Medicine, Baltimore, MD 21201, USA

**Keywords:** Alzheimer’s disease, amylin, amyloid β, molecular dynamics simulation, machine learning

## Abstract

Amylin and amyloid β belong to the same protein family and activate the same receptors. Amyloid β levels are elevated in Alzheimer’s disease. Recent studies have demonstrated that amylin-based peptides can reduce the symptoms of Alzheimer’s disease in animal models. Replica exchange molecular dynamics simulation machine learning, as well as other computational analyses, were applied to improve the understanding of the amino acid residues in these amylin-based peptides. Comparisons were made between amylin, amylin-based peptides, and amyloid β. These studies converged on amylin residues 10Q, 28S, 29S, 30T, 31N, 32V, 33G, 34S, and 35N (residues 10 and 28–35) being ranked highest, meaning that they were the most likely to be involved in activating the same targets as amyloid β. Surprisingly, the amyloid β signaling domain most closely matched amylin residues 29–35 in the simulated structures. These findings suggest important residues that are structurally similar between amylin and amyloid β and are thus implicated in the activation of the amylin receptor.

## 1. Introduction

Alzheimer’s disease is the most common form of dementia [1,2]. In 2024, an estimated 6.9 million people are living with Alzheimer’s disease in the United States, according to the Alzheimer’s Association [3]. Excessive amounts of amyloid β have been observed in the brains of Alzheimer’s patients with the oligomerization of peptides forming clumps outside of cells called plaques [4]. Some studies have suggested that amyloid β oligomerization and plaque formation play a critical role in the progression of Alzheimer’s disease by interfering with neuronal function, while other studies have suggested that the signaling role of amyloid β is more important [4,5].

Amyloid β peptide signaling involves binding to molecular targets in the cell, such as the amylin receptor, calcitonin receptor, and CGRP receptor [6]. The activation of the amylin receptor via either amylin or amyloid β activates a G protein signaling cascade that mobilizes intracellular Ca^2+^ release and triggers signal transduction pathways that can ultimately result in apoptotic cell death [7]. Amyloid β and amylin induce apoptosis through the activation of the amylin receptor, which can be blocked by antagonists of the amylin receptor [8]. Such activation of the amylin receptor, which is a class-B GPCR, also results in the mobilization of intracellular Ca^2+^ and the activation of protein kinase A, MAPK, Akt, and cFos [9].

There have been several studies suggesting that amyloid β toxicity arises through signaling via the amylin receptor [10]. Other studies have indicated that amylin and amyloid β form amorphous heterocomplexes that increase neural toxicity and promote cell death over amylin or amyloid β alone [11]. Paradoxically, Zhu and co-workers found that patients with very low or very high blood levels of amylin had a decreased incidence of Alzheimer’s disease [12]. Zhu and co-workers also found that the intraperitoneal injection of pancreatic amylin reduced behavioral impairment in a mouse model of Alzheimer’s disease [13]. Jhamandas and MacTavish have shown that the amylin antagonist AC187 attenuates the activation of initiator and effector caspases that mediate Aβ-induced apoptotic cell death in rats and the expression of apoptotic genes [8,14]. More recently, Soudy and co-workers have shown that an amylin receptor-enriched extracellular vesicle can bind soluble oligomers of amyloid β to protect neuronal cells against the toxic effects of this peptide [15].

There have been many efforts to develop drugs for the treatment of Alzheimer’s disease. Many of the failed drugs for Alzheimer’s disease have targeted the formation or oligomerization of amyloid β [5]. These drugs include semagacestat, avagacestat, tarenflurbil, lanabecestat, verubecestat, atabecestat, bapineuzumab, solanezumab, gammagard liquid, LMTM, and ponezumab. However, drug-targeting oligomerization has failed to improve disease symptoms or progression. In contrast, the three drugs that have received FDA approval in the United States target the amyloid β signaling domain (gantenerumab targeting residues 3–11 and 18–27, aducanumab targeting residues 3–7, and donanemab, a drug that targets amyloid β residues 3–7) [5,16]. Residues 3–7 lie in the oligomerization domain, and residues 18–27 are in the oligomerization domain. Instead of the small molecules and antibodies proposed above, short peptides have been proposed as a possible treatment for Alzheimer’s disease [17,18,19]. For example, amylin receptor antagonist peptides have been shown to address memory deficits in an Alzheimer’s disease mouse model by blocking the neurotoxic effects of amylin receptor activation [20].

Amylin and amyloid β have both been shown to activate the amylin receptor. Receptor activation happens when the ligand binds to the ligand binding site on the receptor. This requires that the ligand fits in the binding site and interacts with important residues in the site. For this to happen, the ligand must have structural similarity (shape) and sequence similarity so that residues in key positions have similar physicochemical properties. Studying the structural and sequence similarities between amylin and amyloid β through molecular simulation can provide some insight into how they interact with the receptor. This study used molecular simulation and computational analysis to address the key question of what residues are structurally similar in peptides that bind to the amylin receptor and are thus important in activating the amylin receptor. Being able to bind but not activate the amylin receptor is important for drugs that can address Alzheimer’s disease. The study first identified the amino acid residues of the amylin peptide that play a role in the agonism or antagonism of the amylin receptor. This study will explore structural similarities between amylin and amyloid β to understand how they both can signal the same receptors.

## 2. Materials and Methods

### 2.1. Replica Exchange with Solute Tempering (REST2) Simulation of Amylin and Amyloid β

**Figure 1:**
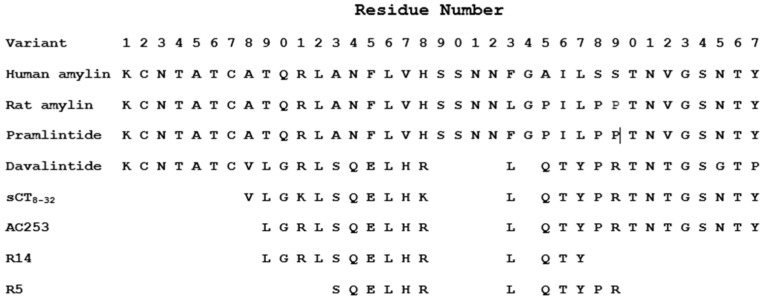
Amylin-based peptides. According to Soudy and co-workers, the peptides are listed from those with the greatest agonist activity at the top to those with the greatest antagonist activity at the bottom [20].

The PDB NMR structures for amyloid β (1IYT) and amylin (2L86) were used for the starting structure for the wild-type simulations. Amylin-based peptides were chosen from Soudy et al. for the REST2 simulation [20]. These peptide sequences are shown in Figure 1. During our modeling pipeline, before REST2 was applied, we subjected these structures to MD simulations at 700 K until all of the secondary structure was eliminated. As a result, the precise peptide poses found in the PDB structures were not expected to affect our results.

#### 2.1.1. Template Creation

In these studies, a single amylin or amyloid β peptide in water was modeled using the CHARMM36m force field and capped with acetylated and amidated N- and C-termini, respectively [21]. CHARMM36m was chosen based on previous studies demonstrating that it performs well for intrinsically disordered peptides [22,23]. System solvation was accomplished with CHARMM-modified TIP3 water molecules and with counterions to neutralize the peptide net charge. Molecular dynamics simulations were run with the program NAMD [24,25,26]. The NAMD parameters followed those used by our group [5,27]. Briefly, MD simulations utilized a 1 fs integration step with full electrostatic interactions and van der Waal interactions smoothly switched off in the interval from 8 to 12 Å. The temperature was controlled with underdamped Langevin dynamics, and the damping coefficient was set to 5 ps^−1^. The pressure was held constant at 1 atm via the Langevin piston method. The covalent bonds linking hydrogen atoms were constrained with the ShakeH algorithm.

#### 2.1.2. REST2 Simulation

**Figure A1:**
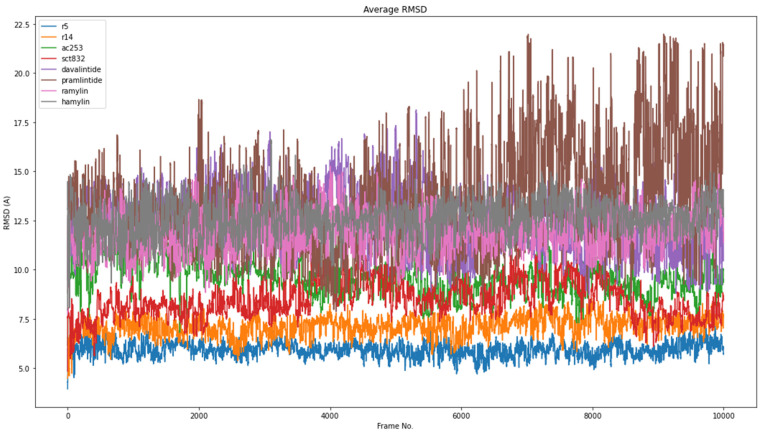
The RMSD deviation levels off for the REST2 simulations, indicating that the simulations have converged.

REST2 simulations were carried out using the replica exchange TCL scripts distributed via NAMD (https://www.ks.uiuc.edu/Research/namd/, last accessed on 5 August 2022). In total, R = 8 replicas for R5, R14, AC253, and sCT_8-32_ and R = 12 replicas for davalintide, pramlintide, rat amylin, and human amylin were simulated with temperatures geometrically distributed from 310 K to 510 K. Exchanges were attempted every 2 ps, and the simulations were run for 20 ns per replica in a trajectory. The corresponding acceptance rates for amylin and amyloid β simulations were 20% and 11%. For each peptide, 3 REST2 trajectories were generated, resulting in a total simulation time of 0.48 μs or 0.72 μs per peptide. We used coil-only structures from our pre-REST2 simulations as starting points for the REST2 simulations. This ensured unbiased conformational sampling, allowing the system to explore a wide range of structural states. The REST2 deviation of the simulated peptides leveled off, indicating the convergence of the simulations, as shown in Figure A1. REST2 simulations were performed on Exxact Valence workstations with a 64-core AMD Threadripper 3995WX and 4 NVIDIA RTX A6000 GPUs.

### 2.2. Comparison of Amylin and Amyloid β PDB Structures

#### 2.2.1. Clustering of Similar Structures

The structures for human amylin and wild-type amyloid β obtained from the REST2 simulations at 310 K were clustered using the algorithm by Daura et al., which works as follows [28]. A representative structure is first determined by finding the structure with the greatest number of neighbors (over 2%) within the Cα RMSD cutoff (set to 2 Å to produce a collection of structurally similar clusters). This representative structure and its neighbors are then set aside as a cluster and removed from the pool of available structures. These steps are repeated until all structures are assigned to a cluster. To generate the pool of available structures, the last 1000 structures at 310 K from each REST2 trajectory were extracted using VMD. This procedure yielded 4 clusters for human amylin and 7 clusters for wild-type amyloid β.

#### 2.2.2. FATCAT Structural Alignment

FATCAT (https://fatcat.godziklab.org/, last accessed on 1 December 2024) was used to structurally align amylin and amyloid β structures [29]. The FATCAT program is also available from the PDB website. The complete peptides were compared using the default settings. The aligned residues with RMSD < 2.0 Å were then tabulated in Microsoft Excel (Redmond, WA, USA) by placing an “×” to count the total number of times the residues were aligned (hits). The mean and standard deviation for the number of hits were calculated. A graded significance approach was employed using the following two criteria: (1) residues with the number of hits exceeding the mean were considered significant, and (2) residues exceeding the mean plus the standard deviation were considered significant.

#### 2.2.3. DOT Plot Alignment

A dot plot was constructed using Microsoft Excel (Redmond, WA, USA). The amylin residues are shown on the vertical axis, and amyloid β is shown across the horizontal axis. The table recorded number of times that there was an alignment of two residues in the 25 FATCAT-aligned structures with RMSD < 2.0 Å.

### 2.3. Feature Extraction from REST2 Trajectories to Form the Data Matrix

These studies used machine learning to see which residue’s dihedral angles can best predict the activation of the amylin receptor. For each amylin-based variant, 3000 structures were chosen from the last 1000 frames in all 3 trajectories. We rationalize this choice below in “Test for Convergence of results”. The frames for all amylin-based variants added up to a 24,000 × 74 object matrix containing the phi and psi angles. This matrix consists of 72 columns for the phi and psi angles, a column for the class, and a column for the variant label. The phi and psi dihedral angles were extracted from the structures. The random forest algorithm was consequently performed on the matrix of the resulting average of angles from the analysis. The TCL code for extracting the phi and psi can be found at the George Mason University Dataverse at https://doi.org/10.13021/orc2020/OW4T3K (accessed on 26 January 2022).

All 3000 structures for each amylin-based variant were labeled with a class designation based on their experimentally determined behavior [20,30,31]. Human amylin, rat amylin, and pramlintide were labeled with the agonist class. The peptides sCT_8-32_ and davalintide were labeled with the low-affinity antagonist class. The peptides AC253, R14, and R5 were labeled with the high-affinity antagonist class. A set of raw data consisting of the dihedral angle for each structure, its name, and its class label was compiled into a matrix and utilized for all machine learning computations.

### 2.4. Machine Learning Analysis of Amylin Structures

#### 2.4.1. Feature Selection

The set of phi and psi angles used in the random forest machine learning model were obtained using feature selection using classification trees. Importantly, the collection of residue dihedral angles provides a comprehensive representation of peptide structure without requiring structure translation or rotation. This was done using the “classification tree” example workflow in the Orange3 software package (https://orangedatamining.com/, accessed on 1 December 2024) with default parameters [32]. The classification tree is part of the random forest methods that create decision trees that can classify the data completely using distinct features (phi and psi dihedral angles). The random forest is made of a collection of trees.

#### 2.4.2. Random Forests

The random forest algorithm was used to predict the classification of each variant. Random forest is a classification algorithm that employs numerous decision trees to separate data classes in the training data. The random forest uses the majority output to decide how to classify a data entry [33]. Machine learning was performed using a random forest algorithm with 5000 trees and otherwise default parameters from Scikit-Learn. We employed a leave-one-out k-fold cross validation (cross validation with k = 1) classification methodology for testing and training. This method uses a test set of 1, the random forest model is trained on the remaining variants’ dihedral angles with the results of the random forest being either an agonist, a low-affinity antagonist, or a high-affinity antagonist. Next, the class of the 1 test structure is predicted either as an agonist, a low-affinity antagonist, or a high-affinity antagonist. Then, another structure is chosen for a test structure, and this is repeated until all individual structures have been evaluated.

### 2.5. Test for Convergence of Results

To ensure that molecular simulations were converged, we first verified that the RMSD deviation for each variant had leveled off (Appendix A, Figure A1). Then, we repeated machine learning and FATCAT analyses on frames 9400–9599, 9600–9799, and 9800–9999 for each trajectory at 310 K. The machine learning results found similar regions of the protein with a slight difference in residues to those sets of 200 frames and the last 1000 frames. On the other hand, if the first 200 frames are used, the angles are more numerous and different. This analysis suggests that the dataset in each trajectory at 310 K represents an equilibrated sampling (Appendix A).

### 2.6. Peptide Sequence Alignment

The sequence alignment of human amyloid β and human amylin was performed using NCBI COBALT with default parameters (available at https://www.ncbi.nlm.nih.gov/tools/cobalt/re_cobalt.cgi, accessed on 1 December 2024). COBALT used pairwise alignment constraints obtained from database searches, sequence similarity, and user input in the progressive multiple alignments it generated [34]. These constraints include the conserved domain database and the PROSITE protein-motif database, which are considered to improve alignment quality. The aligned residues were colored manually (red, blue, and green) to make it easier to see aligned residues. Blue shows exact matches, and green shows residues with similar physiochemical features.

### 2.7. Sorting and Combination of Results

**Figure 2:**
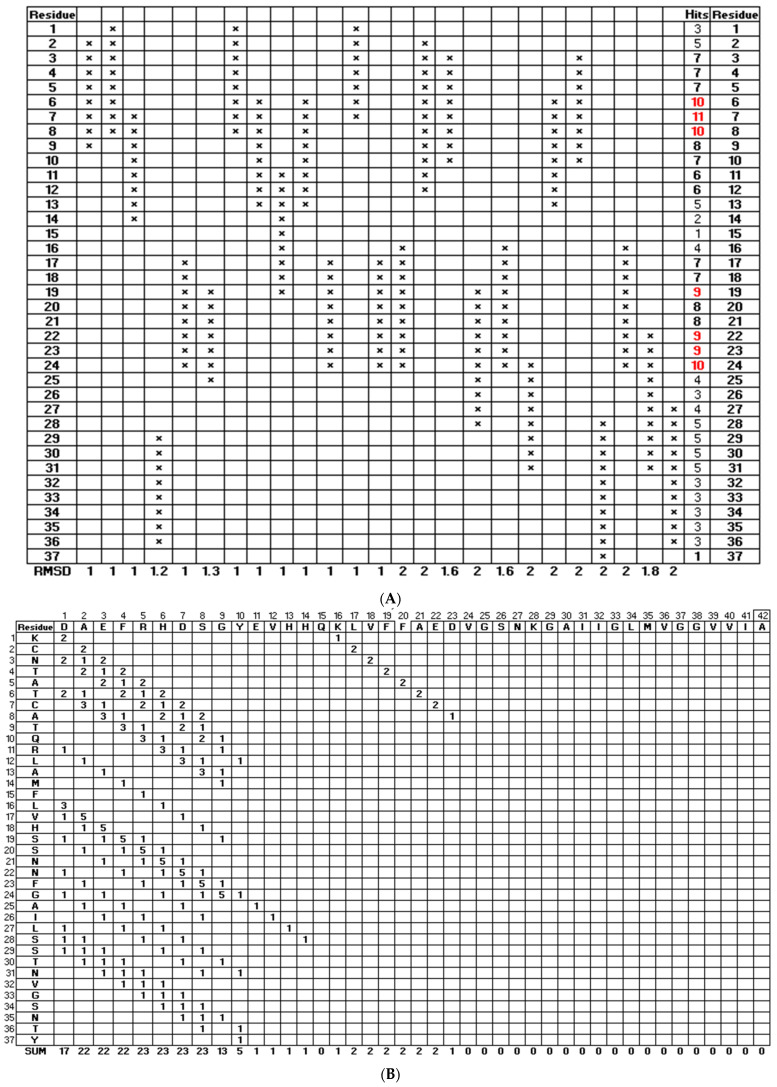
Identification of amylin residues that align structurally with amyloid β. (**A**) Summary of FATCAT structurally aligned residues of amylin and signaling domain (full structure). (Mean = 2.2; standard deviation = 1.8.) Hits are the number of occurrences in the FATCAT structural alignments per position. (Mean = 5.76; standard deviation = 2.7.) Hit values greater than the mean are in bold. Hit values greater than 1 standard deviation above the mean are highlighted in red. The RMSD for the alignments is shown at the bottom. Out of the 28 possible alignments, only those with an RMSD < 2 Å for human amylin compared to wild-type amyloid β were utilized for this analysis. (**B**) Dot plot alignment of human amylin (vertical) compared to wild-type amyloid β (horizontal) for the FATCAT alignments with RMSD < 2.0 Å. The number of times each residue matched between the two sequences is indicated in each box.

The results from the various methods of analysis were combined to understand the most important features (phi and psi dihedral angles). To accomplish this, a table was created in Microsoft Excel (Redmond, WA, USA) with the 37 amylin residues listed on the vertical in the first column. Across the top were the different tests employed. For every residue identified as important via machine learning, a 2 was entered in the table. For the FATCAT alignment analysis shown in Figure 2A, the residues with the hits in red were assigned a value of 2 and the residues with the bolded number of hits a value of 1. Similarly, for the COBALT alignments in Figure 4B, the residues in red were assigned a value of 2 and the blue residues a value of 1. The summation of these values was used to create the data for Figure 5.

## 3. Results

The main goal of this research was to understand which residues of the amylin peptide interact with the amylin receptor and would be likely to compete with amyloid β for amylin receptor binding. Our approach is based on the hypothesis that similar local structures adopted for amylin and amyloid β suggest that both bind to similar regions of the amylin receptor.

### 3.1. REST2 Simulation of Amylin and Amyloid β

The REST2 simulation of amylin peptide and the amylin-based peptides proposed by Soudy and co-workers was used to explore the dynamics of the amylin peptide shown in Figure 1 [20]. To this end, an ensemble of structures was gathered from the last 1000 frames in all three trajectories, yielding 3000 structures for each variant. Each REMD simulation ran for 60 ns to make sure that the system was at equilibrium. The structures were clustered using an RMSD cutoff of 2 Å, yielding 32 clusters from human wild-type amylin and 27 clusters of wild-type amyloid β. Clusters consisting of less than 2% of the structures were discarded, yielding four clusters from human wild-type amylin clusters and seven clusters from wild-type amyloid β obtained from the five replicas at 310 K.

To analyze the ensemble of structures, one representative structure was chosen from each of these retained clusters. FATCAT was used to structurally align amyloid β with amylin (Supplementary Materials S1). Out of the possible comparisons, only the 25 alignments with RMSD < 2 Å were used for the analysis. The alignment results are summarized in Figure 2A, where the aligned residues are indicated with an “×”. The number of aligned residues at each position was summed and reported as “Hits” and those that were aligned in over 50% of the structural alignments are highlighted in red. When the full amyloid β and amylin peptides were structurally aligned amylin residues 3–13 and 17–24 showed hits greater than the average number of hits with residues 6–8, 19, and 22–24 showing the number of hits greater than the mean plus the standard deviation. Figure 2B shows that the FATCAT alignments of the amylin peptide align most closely with (<2 Å RMSD) with residues 2–9 of the human amyloid β peptide. It is important to note that these residues lie in the signaling domain of amyloid β. Amylin residues 6–13 and 16–24 are most likely to align with the amyloid β signaling domain.

### 3.2. Machine Learning Analysis of Amylin Structures

Machine learning was applied to the feature set extracted from the REST2 simulations to characterize the important residues for the antagonistic and agonistic activity of the amylin-based peptides. The phi and psi angles were extracted for 3000 structures from the REST2 simulations for all eight amylin-based peptides shown in Figure 1. The method applied here is an extension of the Molecular Dynamics Phenotype Prediction Method (MDPPM), which takes an ensemble of structures for a collection of proteins, extracts the phi and psi dihedral angles, and uses these as the feature set for molecular dynamics simulations [27,35,36]. To be able to apply the machine learning analysis methods described earlier, called MDPPM, the missing phi and psi angles were imputed by averaging all other measured phi and psi angles for that residue [5,27,35,37].

**Figure 3:**
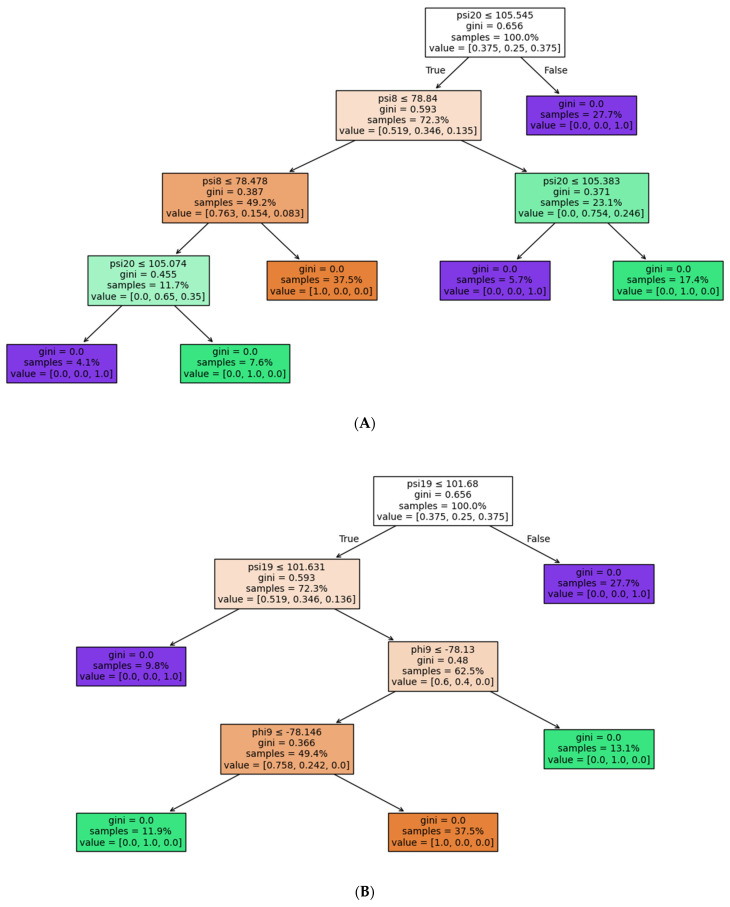

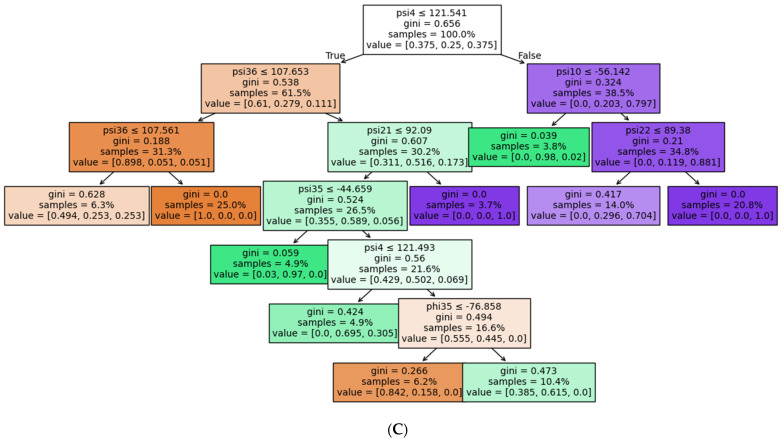
Feature selection using decision trees. (**A**) The first decision tree identified dihedral angles psi8 and psi 20. (**B**) The second decision tree identified dihedral angles phi9 and psi19. (**C**) The third decision tree identified dihedral angles psi4, psi10, psi36, psi21, psi22, psi35, and phi35. Each decision tree node shows the decision assessed at that node, its corresponding Gini coefficient, the percentage of samples subjected to this condition, and the repartitioning of those samples into classes. Node colors indicate the most probable class, where orange represents high-affinity antagonist variants, green represents low-affinity antagonist variants, and purple represents agonist variants.

Feature selection was performed before the random forest machine learning algorithm was applied. To identify these features, decision trees were applied to determine the phi and psi angles that could be used to classify the data, as shown in Figure 3. The number following the phi and psi (e.g., phi20 and psi8) indicates the position in the amino acid sequence with numbering starting from the N-terminal end of the peptide. Classification using decision trees was applied. If the decision tree can perfectly separate the data, we do not impose any threshold. However, if it cannot achieve complete separation, we limit the maximum tree’s leaf nodes to 10 and the minimum leaf sample to 3% to avoid selecting too many angles. Once the first tree determined the classifying angles, those angles were then removed from the feature set. The procedure was repeated, yielding three decision trees and the following dihedral angles: first decision tree: psi8 and psi20; second decision tree: phi9 and psi19; and third decision tree: psi4, psi10, psi36, psi21, psi22, psi35, and phi35.

A classification tree can correctly separate 100% of the variants using phi19, psi8, phi20, and phi9 (Figure 3). The high-affinity antagonist variants are labeled in orange, the low-affinity antagonist variants are labeled in green, and the agonist variants are labeled in purple. A decision boundary is decided by assessing all the possible decision boundaries splitting the dataset and choosing the one that minimizes the Gini impurity of the two splits. Moreover, Gini impurity is a metric that measures the probability of a randomly chosen element (here, a variant) being incorrectly classified, i.e., the probability of choosing an element times the probability of being misclassified. Samples describe the r percentage of variants that need to be classified. Value = [ 0.375, 0.25, 0.375] describes the repartition of these variants among the three possible classes of variants, i.e., the first value is the percentage of the agonist, the second value is the percentage of the low-affinity antagonist, and the final value is the percentage of the high-affinity antagonist. The percentage correctly classified is also shown.

## 4. Discussion

Amylin and amyloid β belong to the amylin protein family or calcitonin/CGRP/IAPP protein family. This family includes the precursors to calcitonin/calcitonin gene-related peptide (CGRP), islet amyloid polypeptide (IAPP), and adrenomedullin, which are in the same protein family and activate the same receptors. Amylin and amyloid β are considered to activate the same receptors, such as the calcitonin receptor, the amylin receptor, and the CGRP receptor [6]. Recent studies have explored the use of fragments of amylin to mitigate the symptoms and progress of Alzheimer’s disease by interfering with amyloid β to the receptor [20]. This study used a series of computational analyses to understand the key amylin residues that trigger signaling responses that overlap with the signaling responses involved with amyloid β. This was accomplished by comparing the structure and sequence of amylin with amyloid β. Based on our previous studies providing a cumulative ranking of the important residues presented here, drugs that target amyloid β residues 27–29 and/or 2–8 will be effective in reducing the symptoms of Alzheimer’s disease [5]. This is consistent with the first 10 residues that have been recognized at the signaling domain of amyloid β. Therefore, this study also compared the first 10 residues of amyloid β with amylin peptide.

Experimental studies have partially addressed which residues are important. In experiments, amylin residues 1–8 have been shown to reduce gastric acid section in rats, but with reduced potency [38]. Similarly, cyclized fragments of amylin residues 1–8 have shown a very reduced ability to stimulate rat neonatal osteoblast activity [39]. However, the C-terminus is necessary for full biological activity [40]. In experiments that systematically substituted an alanine for each residue in amylin, one at a time, substitution at residues 6, 12, 30, 31, and 32 resulted in the most significant reductions in activity [41]. Mutation Y25P of the antagonistic amylin analog AC413 affected interaction with the RAMP2 extracellular domain of the calcitonin receptor [42]. This might suggest that amylin residue 26 is important for the interactions between amylin and the amylin receptor.

**Figure 4:**
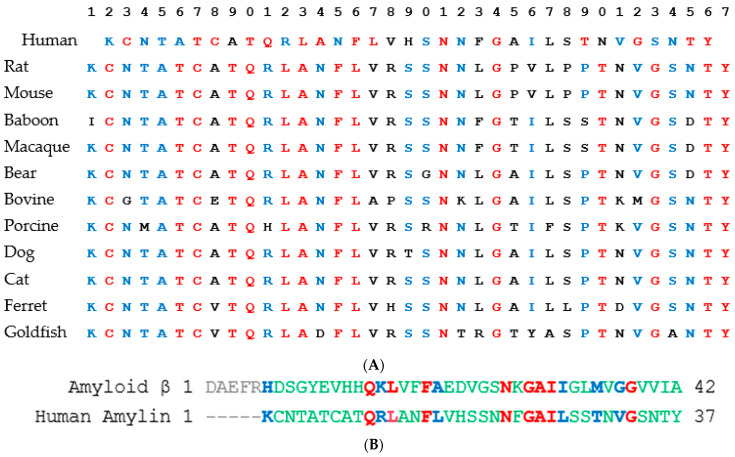
(**A**) Multiple sequence alignment of amylin from different species reproduced using the sequences and alignment from Bower and co-workers [40]. (**B**) Alignment of human amyloid β and human amylin was performed using NCBI COBALT with default parameters. The aligned residues are shown in color (red, blue, and green). Blue shows exact matches, and green shows residues with similar physiochemical features.

Homology studies of amylin show a high degree of conservation across species; see Figure 4A [40]. The residues shown in red are highly conserved, and those in blue are also conserved across species. It is important to note that residues G24, T30, G33, and T36, as well as the C-terminal aromatic Y37, are highly conserved across species [40]. Furthermore, residues 26 (I or V) and 32 (V) are small and nonpolar, and P is often residue 29, but in humans and primates, they are substituted with an S [40]. Experiments with calcitonin, which is in the same protein family as amylin, have shown that the affinity for the receptor remained high with mutations in residue 22–32 and mutations N26D, S29P, and P32HYP increasing affinity [43]. Therefore, other analyses are needed to identify the most important residues for signaling.

Previous studies have suggested that amyloid β peptide and calcitonin signaling involve binding to the same receptors, such as the amylin receptor, calcitonin receptor, and CGRP receptor [6]. It is, therefore, important to understand the structural similarities between the two peptides. A pairwise alignment of human amyloid β and human amylin was performed using NCBI COBALT with the default settings (Figure 4B). COBALT takes both sequence and structural similarities into account when generating an alignment. Combining the results of the conservation of amylin across species and the similarity between human amylin and human amyloid β yields the following residues of amylin: K1, Q10, R11, L12, F15, L16, N22, G24, I26, T30, V23, and G33.

Because human amyloid β and human amylin are largely unstructured, REMD simulations were performed to collect an ensemble of conformations [6,40]. FATCAT alignments of amyloid β and human amylin of <2 Å RMSD aligned amylin residues 3–13 and 17–24 showed hits greater than the average number of hits with residues 6–8, 19, and 22–24 showing the number of hits greater than the mean plus the standard deviation (Figure 2). These residues aligned most closely with residues 2–9 of human amyloid β, which is the signaling domain. Figure 1 shows that the removal of these amylin residues is critical in the creation of peptide variants that can block amylin receptor activation via the peptide. This contrasts with previous studies that have called attention to residues 7–17 of both peptides, which are thought to form an amphipathic helix in their analysis [40,44].

There are several choices for the starting amylin structure in the scientific literature. The methods used in this work have been shown to predict the functional consequence of variants in isolated proteins [5,27,37]. The freely solvated structure assumes many conformations that have been sampled with the REST2 simulation capturing the structural dynamics. This is critical for amylin’s approach and binding to the receptor. Another possibility is the recently produced Cryo-EM structure of rat amylin bound to the amylin receptor by Cao and co-workers [45]. In their structure for amylin, the first 23 residues are highly resolved and inserted into the receptor. However, the C-terminus is of a lower resolution and has a less well-defined structure. Once put in solution and simulated with REST2, it would converge on structures similar to those that were obtained.

Machine learning was applied to the phi and psi angles extracted from REST2 simulations of amylin and the amylin-based peptides. Each variant was assigned a class labeled as an agonist, a low-affinity agonist, or a high-affinity antagonistic. Values for the missing phi and psi angles due to deletions in the amylin-based peptides were imputed by averaging the other entries. The random forests machine learning model chose the dihedral angles that best classified the amylin-based peptides in the appropriate class with 100% accuracy. These angles were phi19, psi8, phi20, and phi9. In other words, the variations in these dihedral angles are different between the three classes. Since structural changes are associated with functional changes, the model suggests that these residues are important for functional differences.

In these studies, random forests (maximum number of trees = 5000 and default parameters in Scikit-Learn) yielded the most accurate predictions. We chose random forests because they model non-linearity and feature interactions and permit the extraction of feature importances. Furthermore, random forests do not overfit as the number of trees increases. Instead, the accuracy stops increasing as the number of trees increases. Also, random forests are robust to noise, and randomness in large datasets can improve classification accuracy, whereas regression, on the other hand, reduces accuracy [33]. We have about 3000 data points showing the variation in phi and psi angles for each of the eight variants. A large amount of data and the exploration of the conformational space (randomness) help explain the robustness of the model. The dihedral angles were identified and used in the random forest machine learning model using leave-one-out validation to maximize the amount of data used in model evaluation. In this validation, the phi and psi angles for the structures of a specified variant would be withheld, and the model was trained on the remaining seven amylin-based peptides. This was repeated for all eight amylin-based peptides. Table 1 summarizes the dihedral angles used and the prediction accuracy.

**Figure 5:**
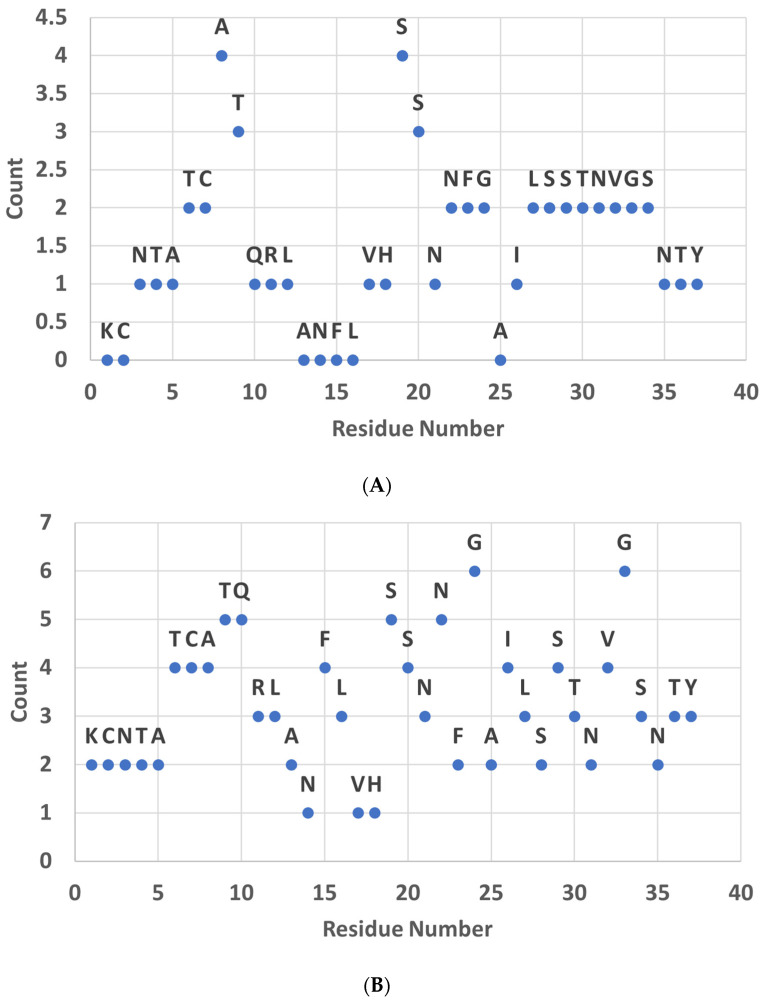
Summary of analysis of significant amylin residues. Counts indicate the number of analyses that indicated that the residue is significant in the different analyses. (**A**) Cumulative summary from analysis of molecular dynamics simulation data. (**B**) Molecular dynamics data analysis combined with information on homology.

The above machine learning predictions were then combined with a count recorded for each analysis that identified the amino acid residues that were identified via the previous methods with structural ensemble alignment to the residues of the signaling domain of amyloid β (residues 1–10) to give a cumulative assessment of the amylin residues involved in governing the agonist/antagonist activity of the amylin receptor (Figure 5). Figure 5A shows measures from analyzing the molecular simulations. A count of one was given each time the residue was identified through each measure. Residues 3–10 and 17–24) ranked in the top half of residue scores (>6), suggesting that they were the most likely to be involved in activating the same targets as amyloid β. Previous studies have suggested that the amyloid β N-terminal signaling domain interacts with other cellular targets [5,46,47]. Bower and co-workers identified four important structural features in rat amylin: (1) the N-terminal disulfide loop; (2) an amphipathic α helix at residues 7–17; (3) an alpha helix at residues 20–24; and (4) a flexible loop at residues 25–37 [40]. In contrast, Soudy et al. found that the deletion of the N-terminal residues 27 and 31–33 leads to a loss of agonist function [20]. It is interesting that in neither this study nor the study by Soudy and co-workers did the C-terminal amino acid residues lead to antagonist function. Figure 5B shows that, when the homology data obtained from sequence alignment is added, the results are more varied, but the same regions still display more hits. These results suggest that the COBALT sequence alignment might not adequately consider the structural effects. This is perhaps because the gathering of the ensemble of structures from the molecular dynamics simulations captures the “dynamics” of the peptide by capturing the different poses it assumes. The COBALT alignment considers a more static structure. This is similar to the case of the MDPPM (this method) when compared to the tools predicting the pathogenicity of the protein/peptide variant (PredictSNP, MutationAssessor, Polyphen, etc.) [27,35]. Those tools consider the PDB static structure and provide a more variable and lower accuracy of prediction than the MDPPM, which captures dynamics.

In addition to the amylin peptide fragments studied (Figure 1), other peptide drugs have been proposed in the efforts to alleviate Alzheimer’s disease. Several peptides containing KLVFF (residues 16–20 of amyloid β), with the names Aβ(16–20), Aβ-(15–22), Aβ-(16–23), Aβ-(17–24), K4, OR1, OR2, and Aβ12–28P, have been shown to inhibit amyloid β aggregation [48]. AMY-1, AMY-2, P1, and P2 (all of which contain KLVFF), as well as Aβ16–22m and Aβ16–20m, have been shown to prevent fibril formation [48]. The R8-Aβ(25–35) peptide was shown to reduce Aβ amyloid accumulation and reduce memory deficits in a transgenic mouse model [49]. These correspond to regions of similarity in the COBALT alignment (Figure 4B) but are not identified via molecular simulation or machine learning (Figure 5). It is important to note that none have made it through clinical trials, and the model was not trained on amyloid β peptide fragments.

When interpreting the study findings, it is important to consider study assumptions and limitations. Our REST2 simulations evaluating the structures of amylin were conducted in explicit solvent. The major assumption of our simulations was that the structures observed in water are relevant to its association with the amylin receptor. We believe that, when a ligand approaches a receptor, its conformational movements govern its first contact and capture via the receptor. Our study was based upon the hypothesis that the structural ensemble can inform us indirectly of these dynamics and accessible states for amylin receptor binding, and this can be used to predict the outcome of amylin variants. Similar hypotheses were made in our previous papers, and we found that our machine learning models are predictive of phenotype from simulations [5,27,35,37].

## 5. Conclusions

In this study, a replica exchange molecular dynamics simulation was combined with machine learning and other computational analyses to identify the amino acid residues in these amylin-based peptides that activate the same targets as amyloid β. Comparing amylin, amylin-based peptides, and amyloid β suggested that amylin residues 6–13 and 16–24 are most likely to align with amyloid β signaling domain residues 2–9 and thus are most likely to be involved in activating the same targets as amyloid β. Such an understanding of residues and structures is needed to design peptide drugs that treat Alzheimer’s disease.

## Figures and Tables

**Table 1:** Random forests features and accuracy.

Features	Decision Tree	Accuracy
psi20, psi8, psi19, phi9	1, 2	100.00%
psi20, psi8, psi19, phi9, psi4, psi36, psi10, psi21, psi22, psi35, phi35	1, 2, 3	87.50%
psi20, psi8, psi4, psi36, psi10, psi21, psi22, psi35, phi35	1, 3	75.00%
psi19, phi9, psi4, psi36, psi10, psi21, psi22, psi35, phi35	2, 3	62.50%
psi4, psi36, psi10, psi21, psi22, psi35, phi35	3	62.50%

Decision trees are shown in Figure 3.

## Data Availability

The original contributions presented in the study are included in the article/supplementary material; further inquiries can be directed to the corresponding author.

## Supplementary Materials

The following supporting information can be downloaded at https://www.mdpi.com/article/10.3390/biom15010089/s1: Supplementary File S1, Structural Alignment Results.
