# Supplementary material for: Structural Analysis of Amylin and Amyloid β Peptide Signaling in Alzheimer’s Disease
Source: Biomolecules. 2025 Jan 9;15(1):89. doi: 10.3390/biom15010089 (PMC11763987; doi:10.3390/biom15010089)

Hamylin_2456 – wt_1831:

Entry	Chain	RMSD	TM-score	Identity	Equivalent Residues	Sequence Length	Modelled Residues
hamylin_2456.pdb	A	-	-	-	-	37	37
wt_1831.pdb	A	3.04	0.14	29%	14	42	42

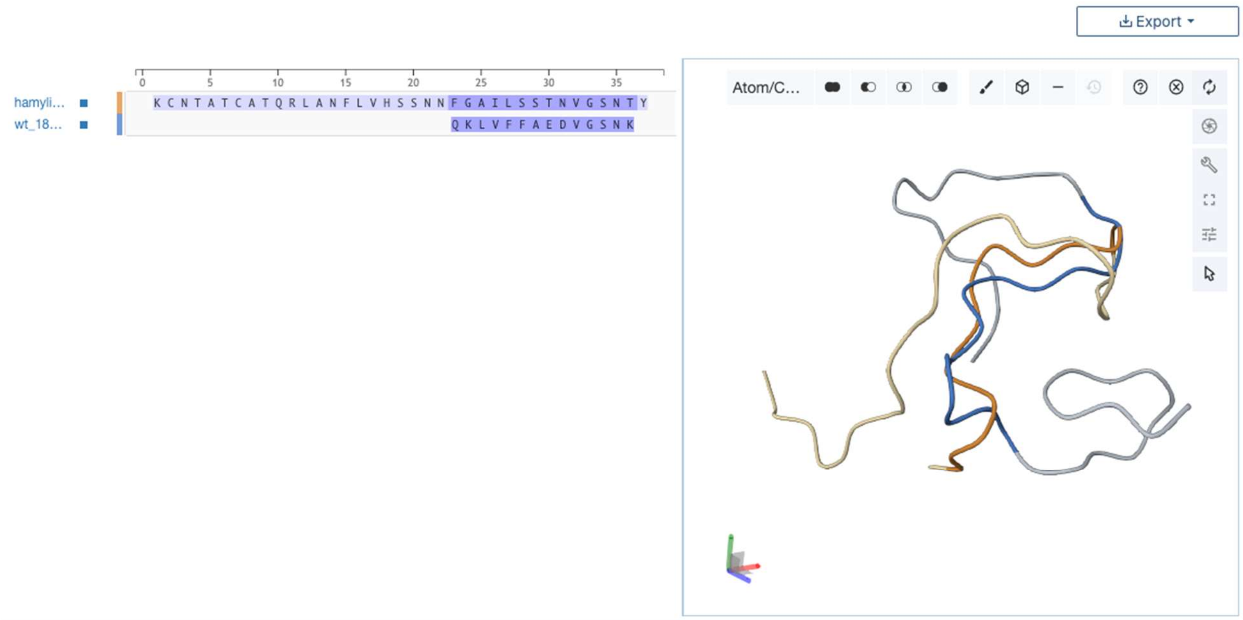

Entry	Chain	RMSD	TM-score	Identity	Equivalent Residues	Sequence Length	Modelled Residues
hamylin_2456.pdb	A	-	-	-	-	37	37
wt_1831.pdb	A	2.15	0.12	10%	10	42	10

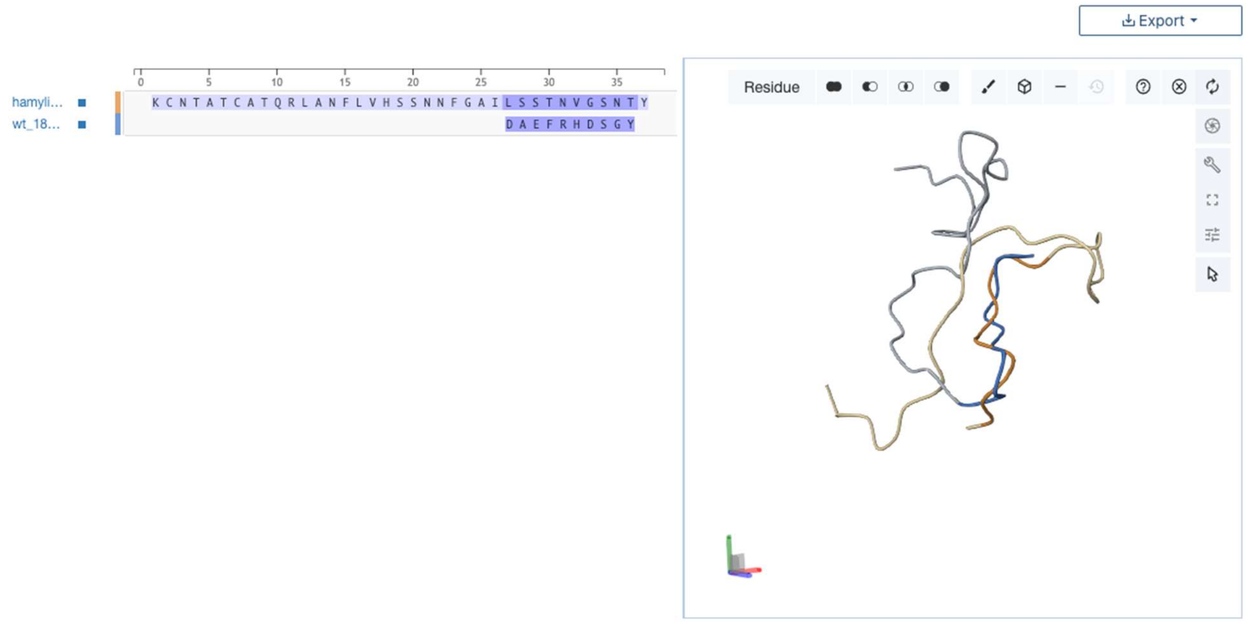

Hamylin_2456 – wt_1334

Entry	Chain	RMSD	TM-score	Identity	Equivalent Residues	Sequence Length	Modelled Residues
hamylin_2456.pdb	A	-	-	-	-	37	37
wt_1334.pdb	A	3.53	0.12	6%	16	42	42

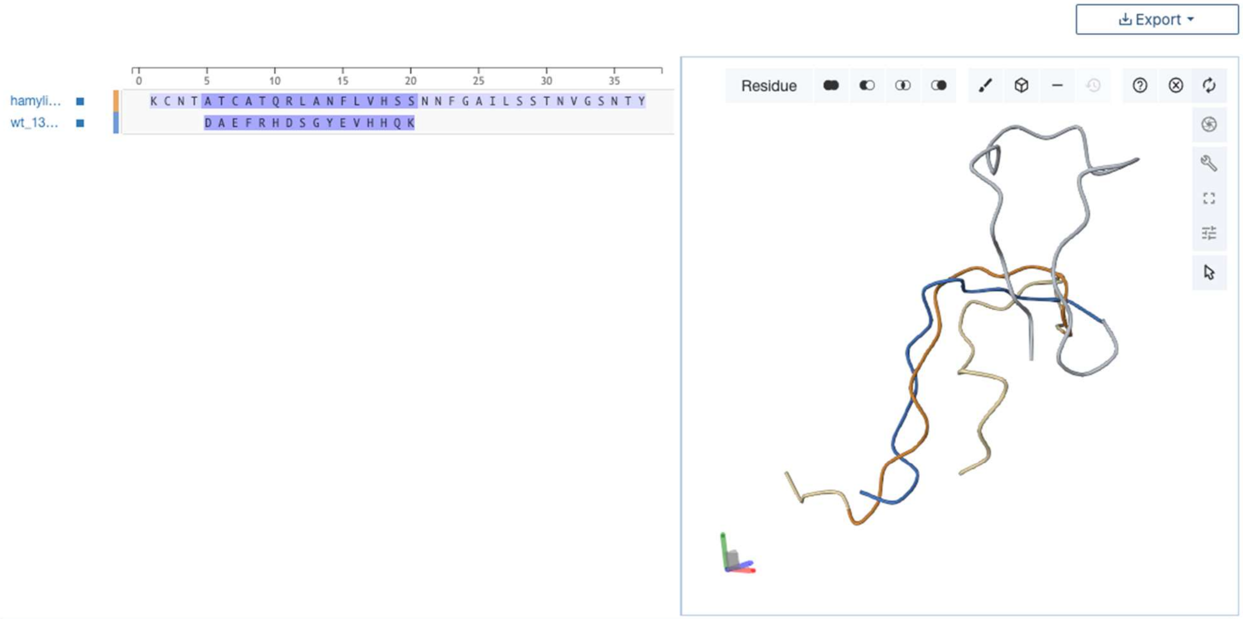

Entry	Chain	RMSD	TM-score	Identity	Equivalent Residues	Sequence Length	Modelled Residues
hamylin_2456.pdb	A	-	-	-	-	37	37
wt_1334.pdb	A	1.36	0.14	0%	8	42	10

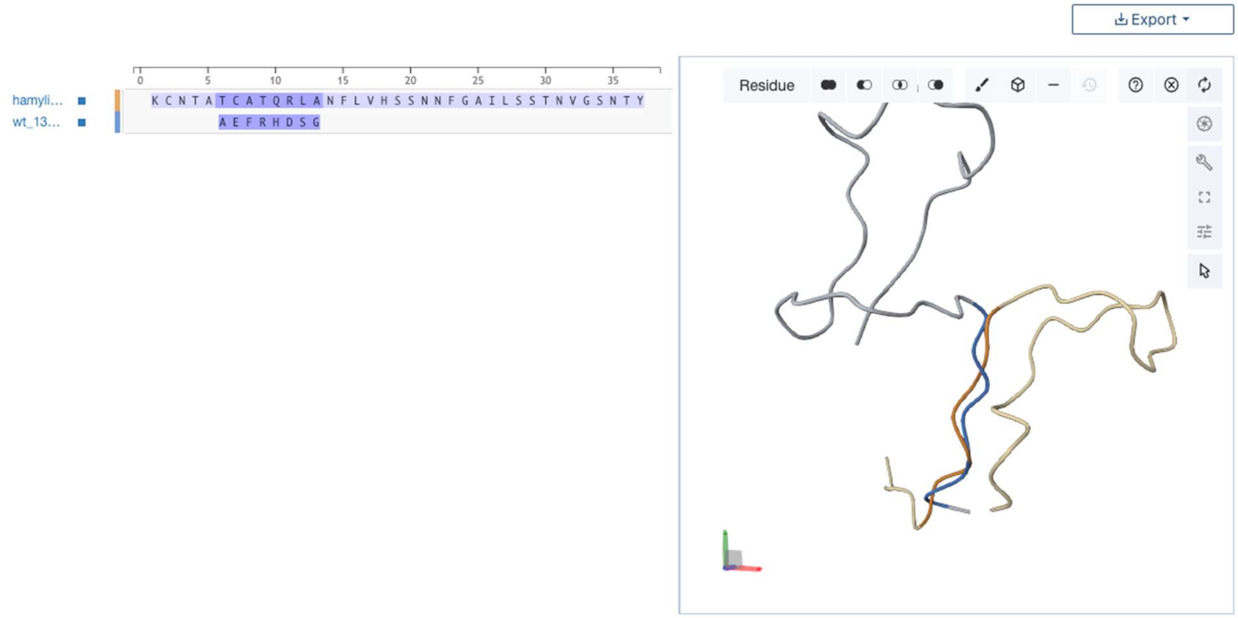

Hamylin_2456 – wt_1159

Entry	Chain	RMSD	TM-score	Identity	Equivalent Residues	Sequence Length	Modelled Residues
hamylin_2456.pdb	A	-	-	-	-	37	37
wt_1159.pdb	A	2.11	0.2	0%	16	42	42

Export

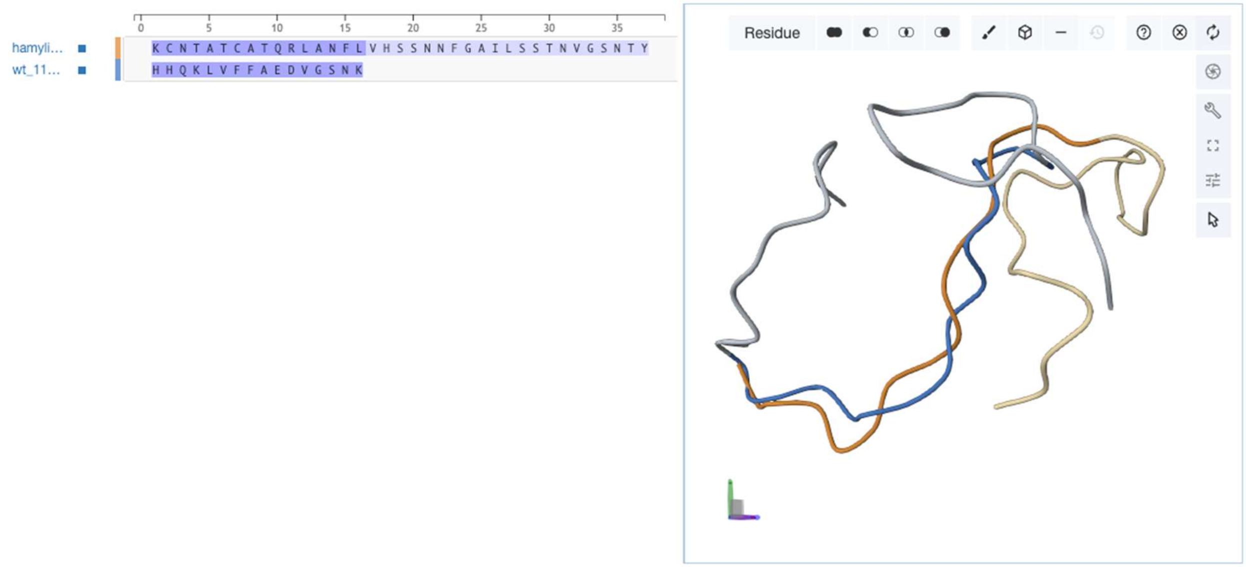

Entry	Chain	RMSD	TM-score	Identity	Equivalent Residues	Sequence Length	Modelled Residues
hamylin_2456.pdb	A	-	-	-	-	37	37
wt_1159.pdb	A	1.36	0.16	0%	9	42	10

Export

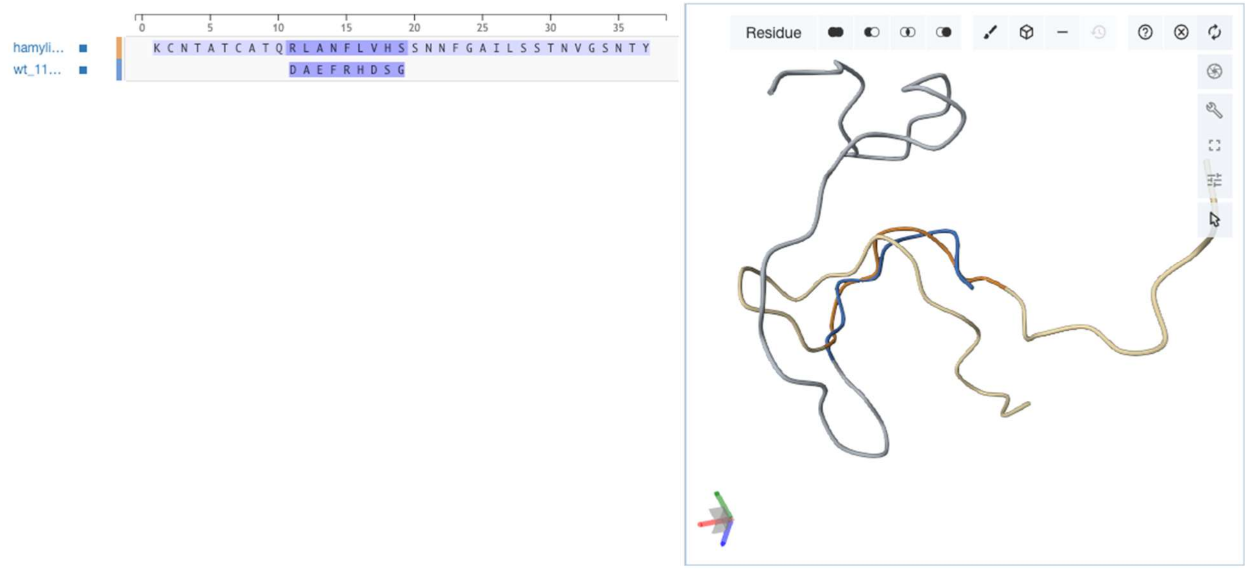

Hamylin_2456 – wt_749

Entry	Chain	RMSD	TM-score	Identity	Equivalent Residues	Sequence Length	Modelled Residues
hamylin_2456.pdb	A	-	-	-	-	37	37
wt_749.pdb	A	3.41	0.21	9%	22	42	42

Export

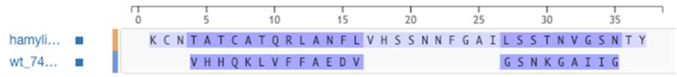

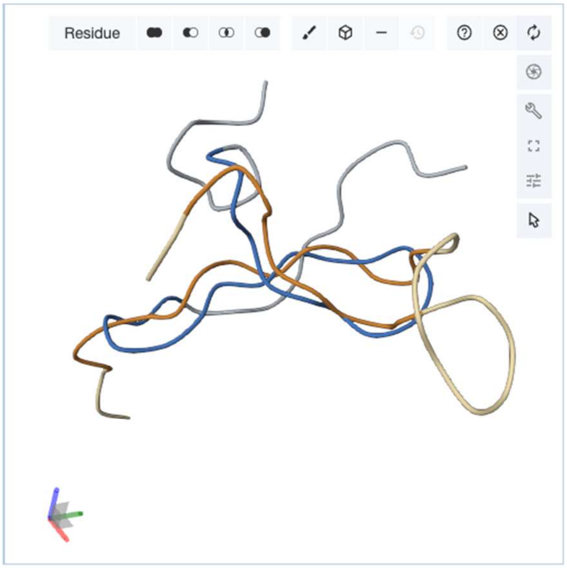

Entry	Chain	RMSD	TM-score	Identity	Equivalent Residues	Sequence Length	Modelled Residues
hamylin_2456.pdb	A	-	-	-	-	37	37
wt_749.pdb	A	1.56	0.17	0%	10	42	10

Export

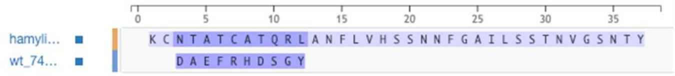

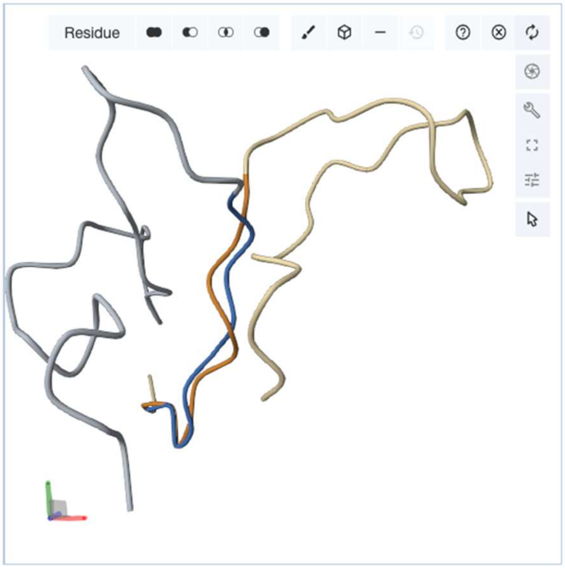

Hamylin_2456 – wt_614

Entry	Chain	RMSD	TM-score	Identity	Equivalent Residues	Sequence Length	Modelled Residues
hamylin_2456.pdb	A	-	-	-	-	37	37
wt_614.pdb	A	6.34	0.07	12%	26	42	42

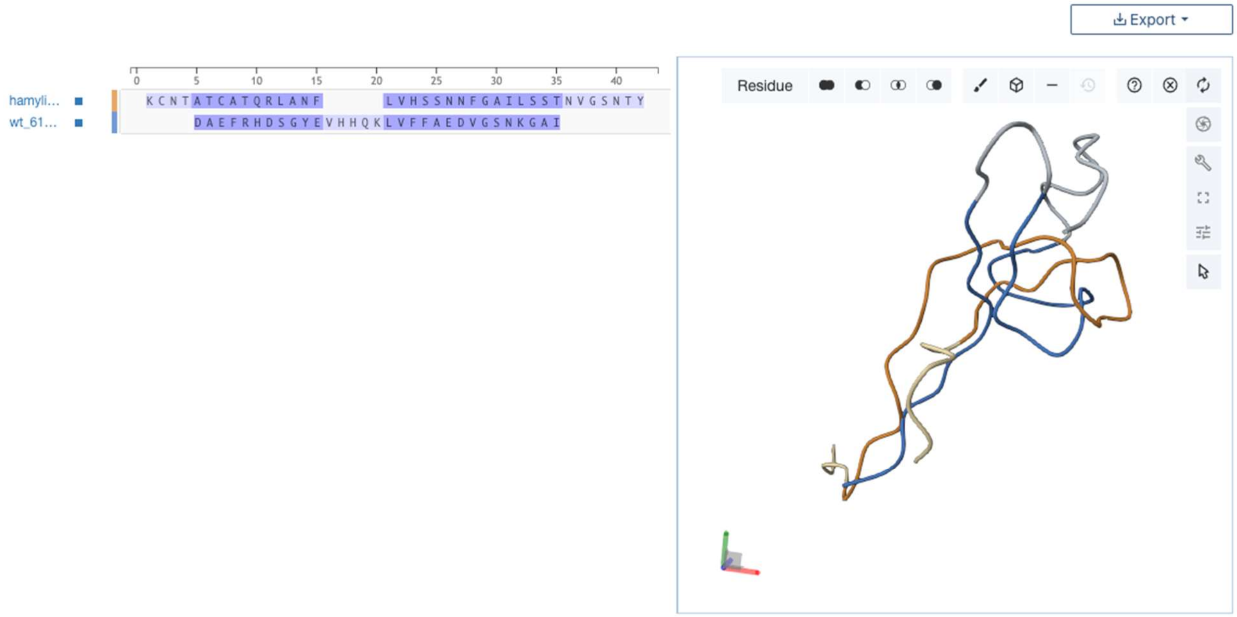

Entry	Chain	RMSD	TM-score	Identity	Equivalent Residues	Sequence Length	Modelled Residues
hamylin_2456.pdb	A	-	-	-	-	37	37
wt_614.pdb	A	1.65	0.12	0%	8	42	10

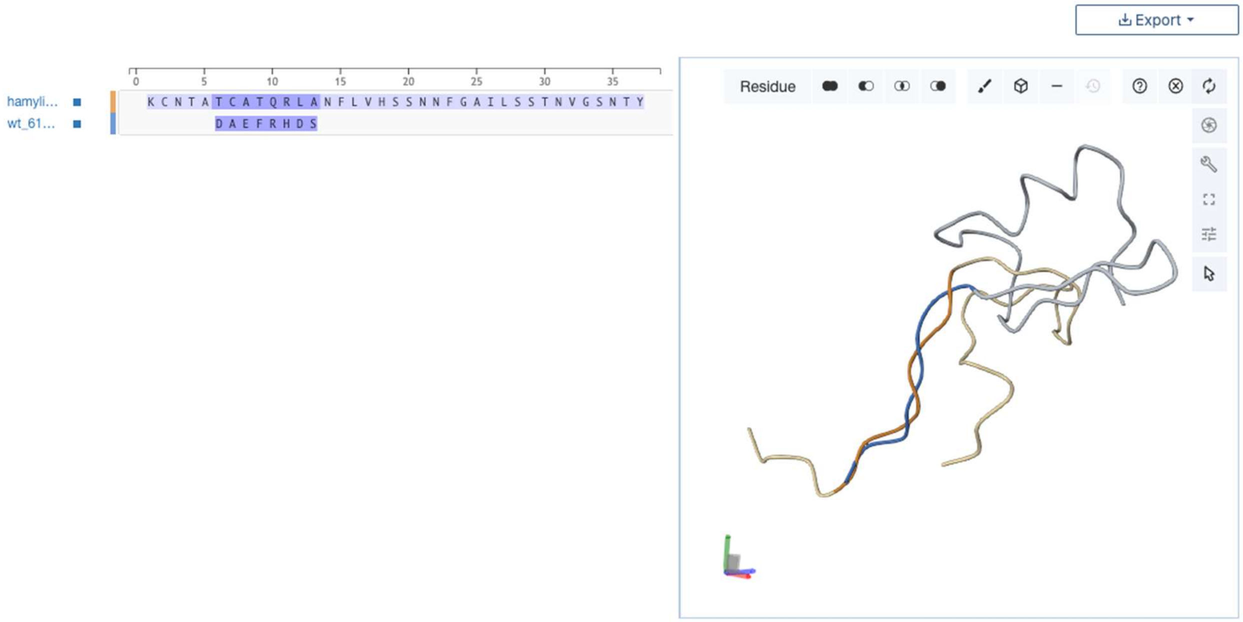

Hamylin_2456 – wt_327

Entry	Chain	RMSD	TM-score	Identity	Equivalent Residues	Sequence Length	Modelled Residues
hamylin_2456.pdb	A	-	-	-	-	37	37
wt_327.pdb	A	3.05	0.15	6%	17	42	42

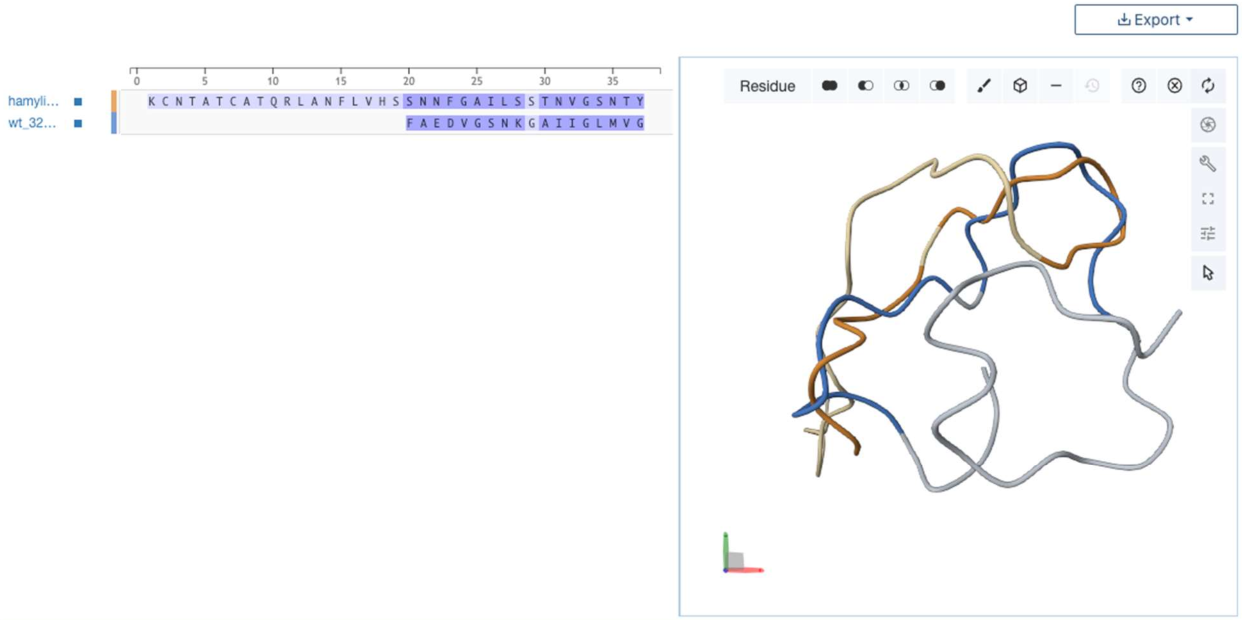

Entry	Chain	RMSD	TM-score	Identity	Equivalent Residues	Sequence Length	Modelled Residues
hamylin_2456.pdb	A	-	-	-	-	37	37
wt_327.pdb	A	1.6	0.13	13%	8	42	10

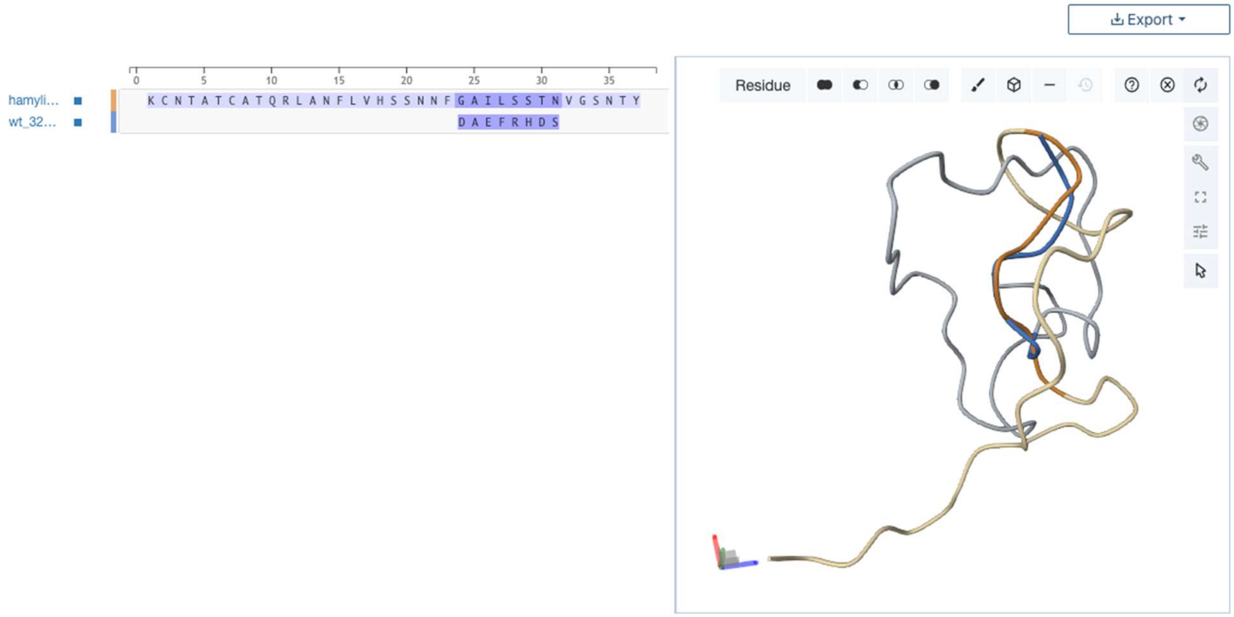

Hamylin_2456 – wt_89

Entry	Chain	RMSD	TM-score	Identity	Equivalent Residues	Sequence Length	Modelled Residues
hamylin_2456.pdb	A	-	-	-	-	37	37
wt_89.pdb	A	3.06	0.17	6%	18	42	42

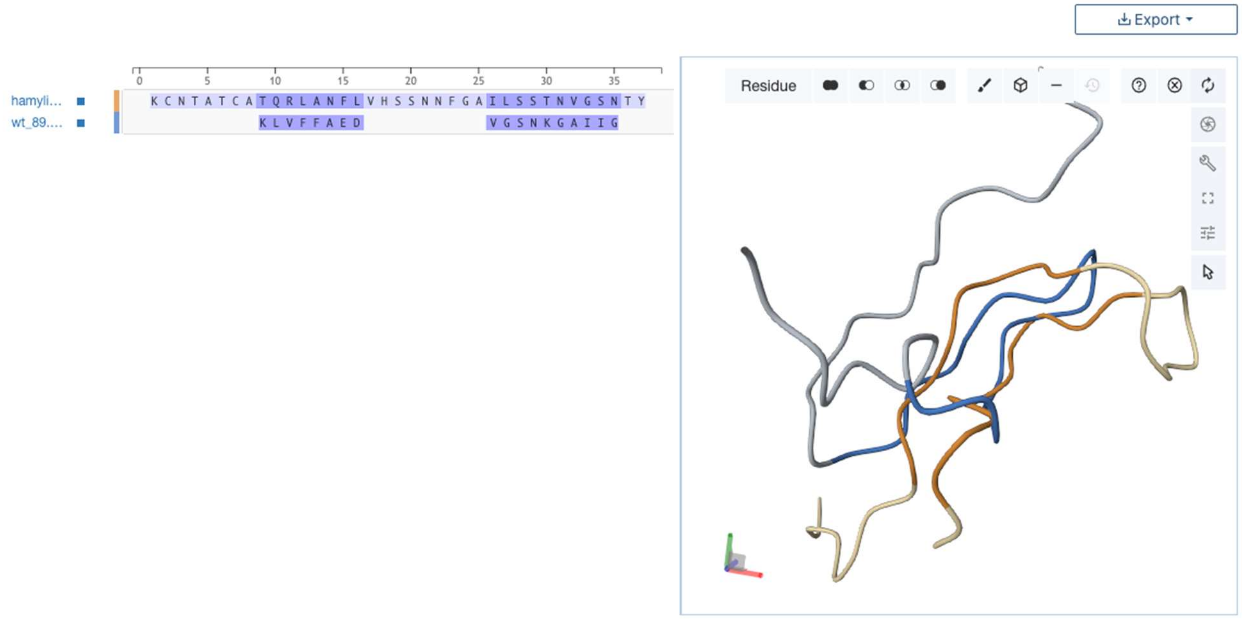

Entry	Chain	RMSD	TM-score	Identity	Equivalent Residues	Sequence Length	Modelled Residues
hamylin_2456.pdb	A	-	-	-	-	37	37
wt_89.pdb	A	1.81	0.14	10%	10	42	10

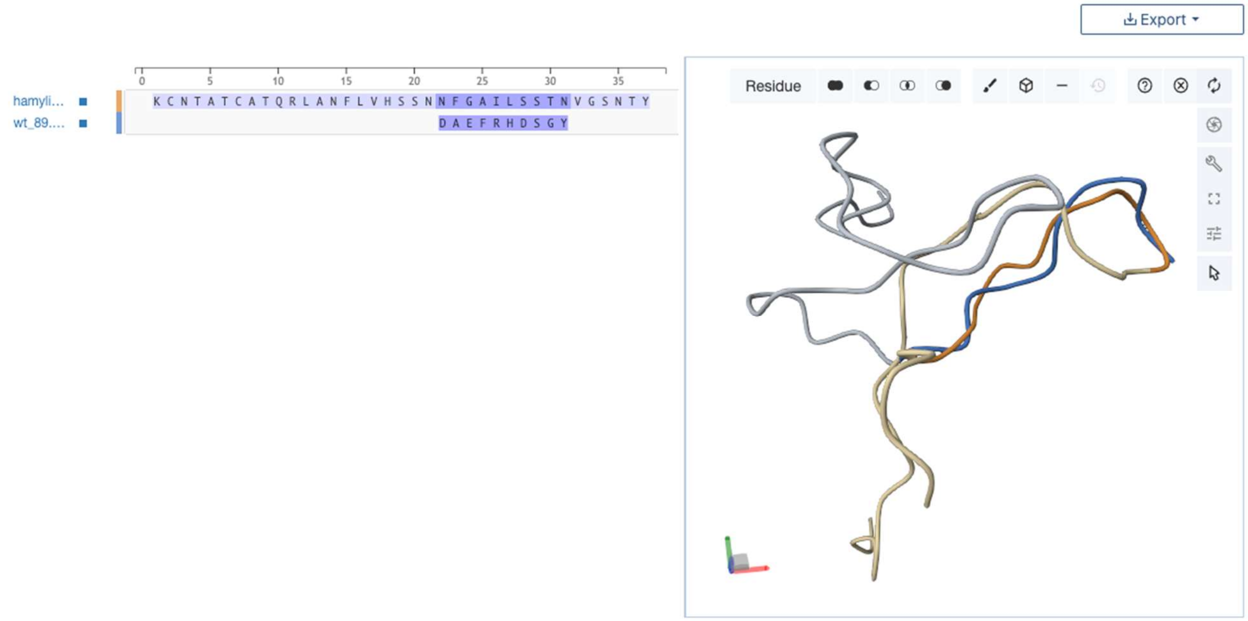

Hamylin_1439 – wt_1831

Entry	Chain	RMSD	TM-score	Identity	Equivalent Residues	Sequence Length	Modelled Residues
hamylin_1439.pdb	A	-	-	-	-	37	37
wt_1831.pdb	A	2.89	0.21	9%	21	42	42

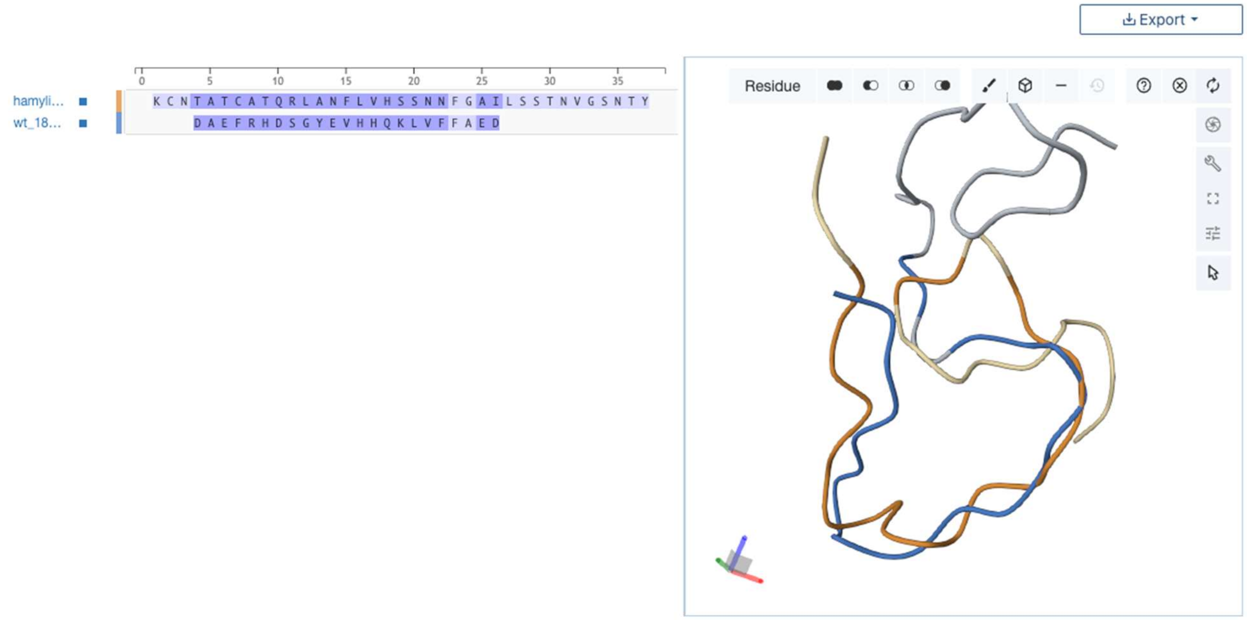

Entry	Chain	RMSD	TM-score	Identity	Equivalent Residues	Sequence Length	Modelled Residues
hamylin_1439.pdb	A	-	-	-	-	37	37
wt_1831.pdb	A	1.42	0.14	0%	8	42	10

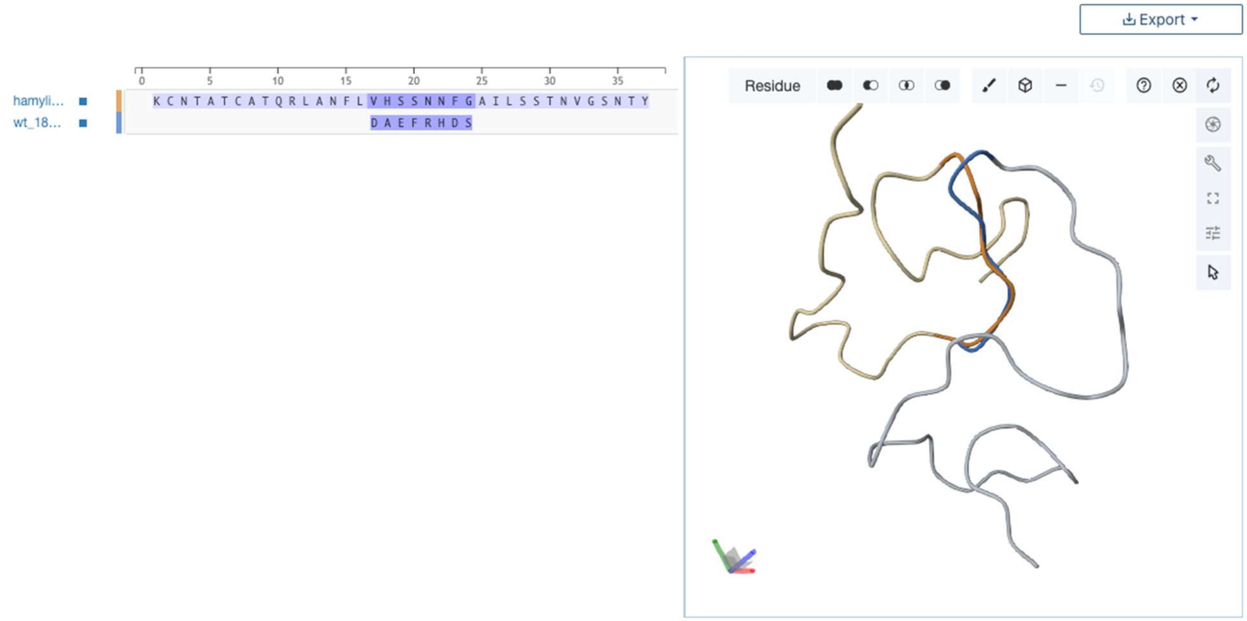

Hamylin_1439 – wt_1334

Entry	Chain	RMSD	TM-score	Identity	Equivalent Residues	Sequence Length	Modelled Residues
hamylin_1439.pdb	A	-	-	-	-	37	37
wt_1334.pdb	A	3.05	0.14	0%	14	42	42

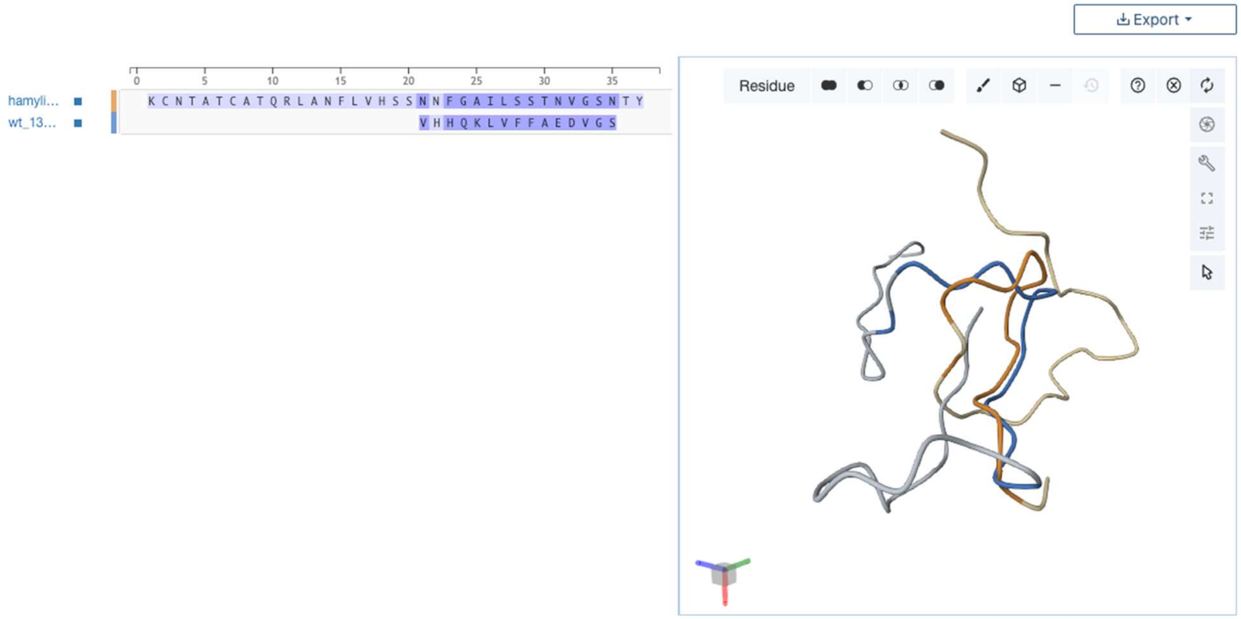

Entry	Chain	RMSD	TM-score	Identity	Equivalent Residues	Sequence Length	Modelled Residues
hamylin_1439.pdb	A	-	-	-	-	37	37
wt_1334.pdb	A	1.2	0.15	13%	8	42	10

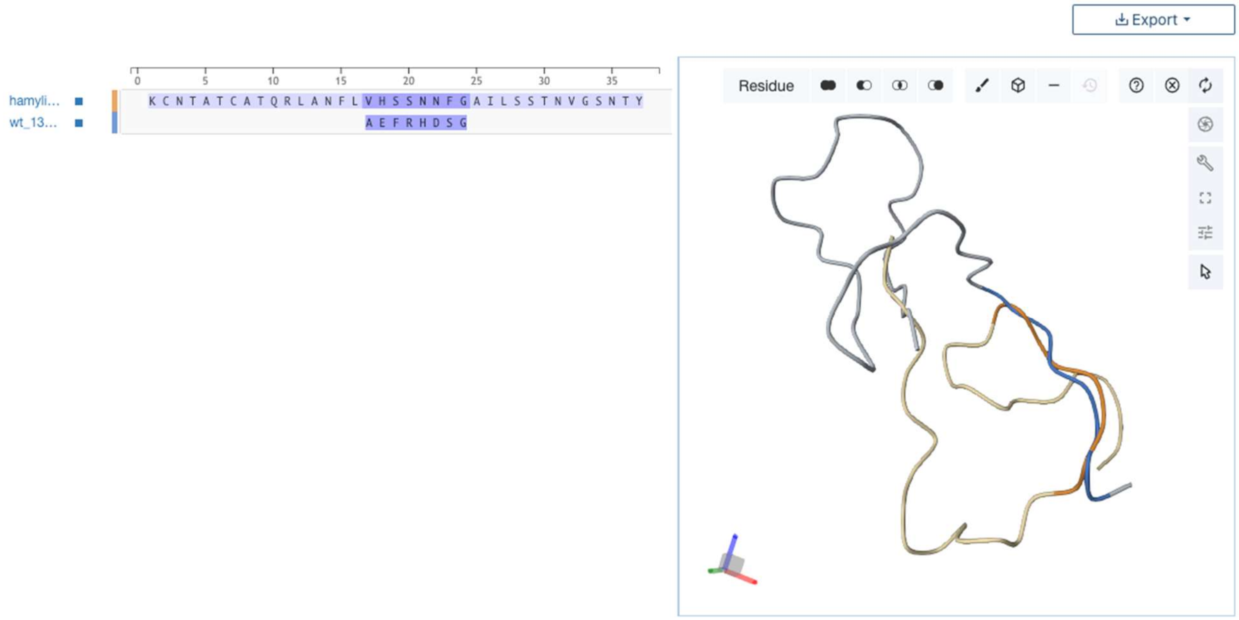

Hamylin_1439 – wt_1159

Entry	Chain	RMSD	TM-score	Identity	Equivalent Residues	Sequence Length	Modelled Residues
hamylin_1439.pdb	A	-	-	-	-	37	37
wt_1159.pdb	A	3.44	0.19	0%	22	42	42

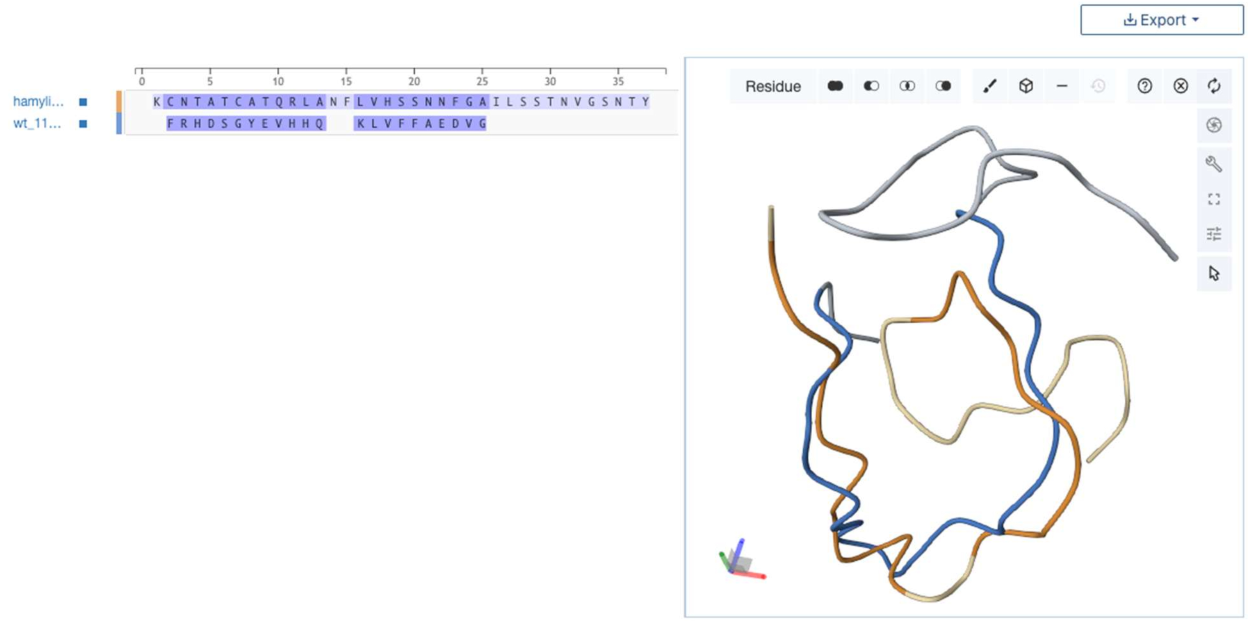

Entry	Chain	RMSD	TM-score	Identity	Equivalent Residues	Sequence Length	Modelled Residues
hamylin_1439.pdb	A	-	-	-	-	37	37
wt_1159.pdb	A	2.79	0.12	0%	10	42	10

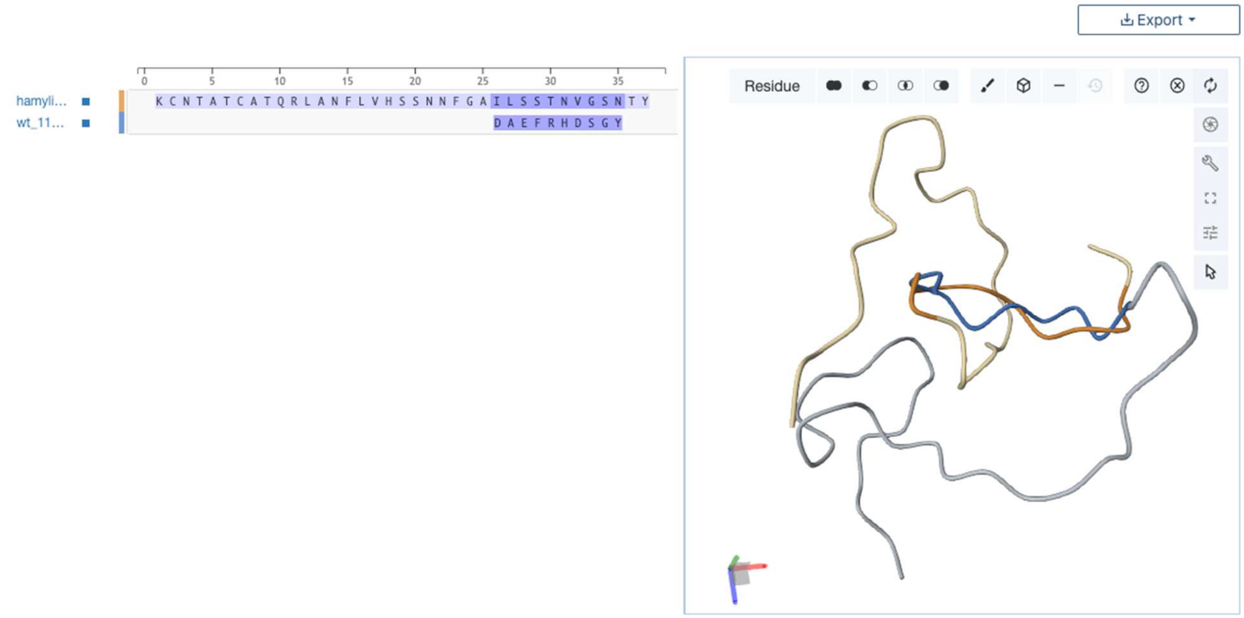

Hamylin_1439 – wt_749

Entry	Chain	RMSD	TM-score	Identity	Equivalent Residues	Sequence Length	Modelled Residues
hamylin_1439.pdb	A	-	-	-	-	37	37
wt_749.pdb	A	3.13	0.19	5%	19	42	42

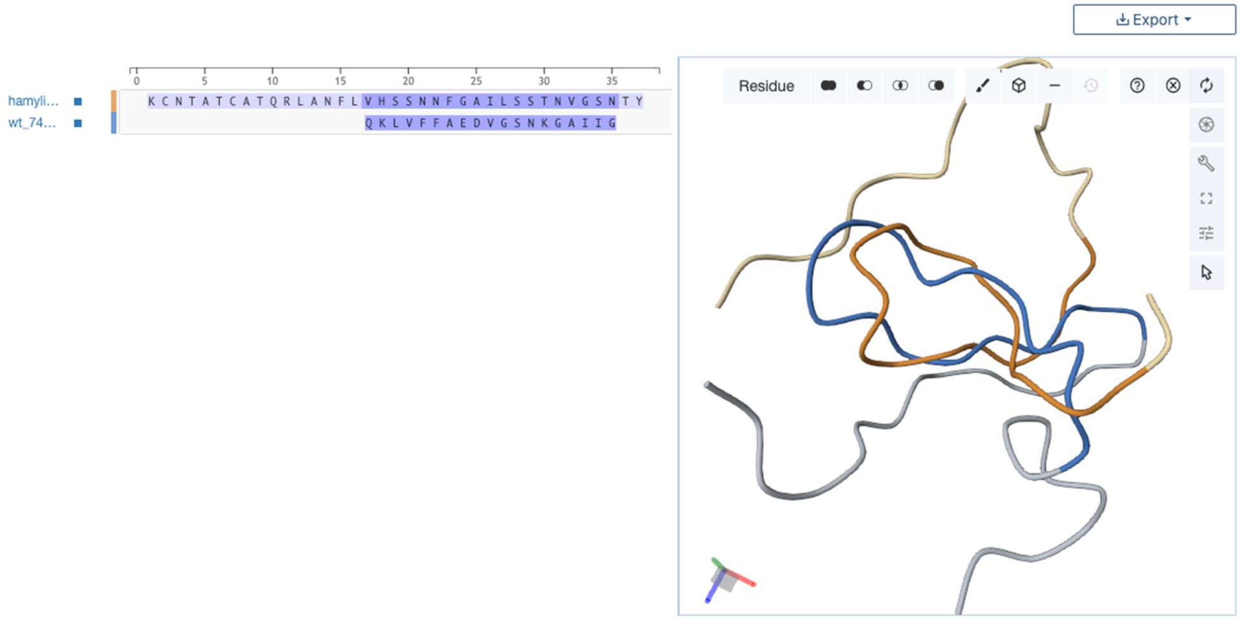

Entry	Chain	RMSD	TM-score	Identity	Equivalent Residues	Sequence Length	Modelled Residues
hamylin_1439.pdb	A	-	-	-	-	37	37
wt_749.pdb	A	1.62	0.13	11%	9	42	10

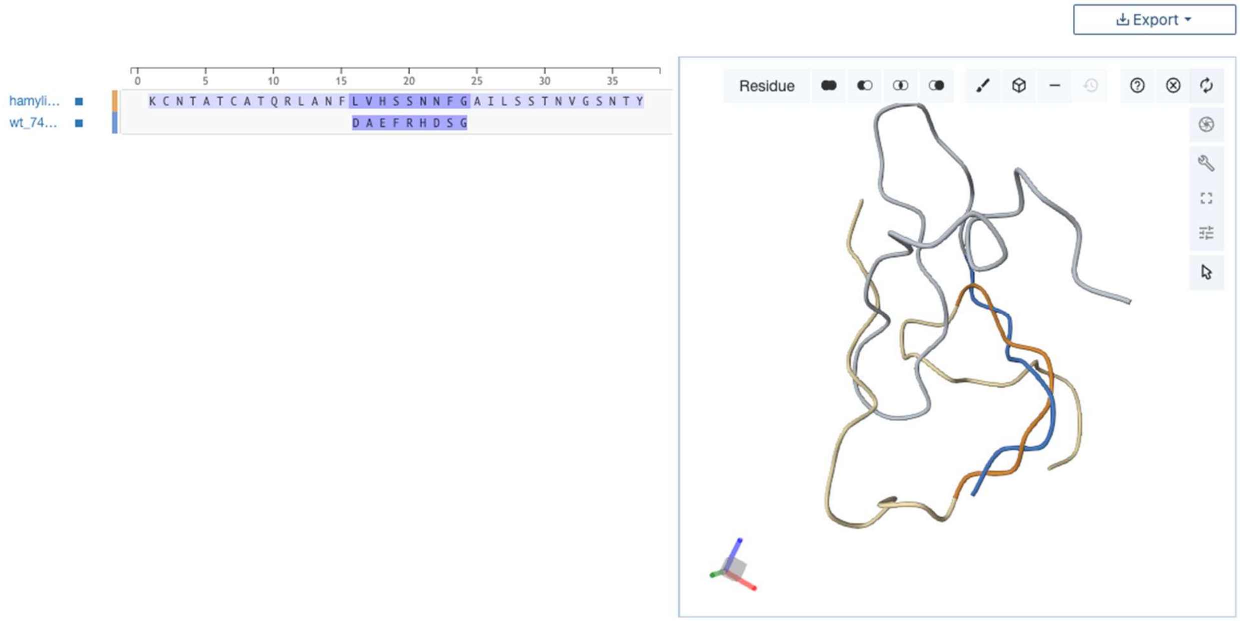

Hamylin_1439 – wt_614

Entry	Chain	RMSD	TM-score	Identity	Equivalent Residues	Sequence Length	Modelled Residues
hamylin_1439.pdb	A	-	-	-	-	37	37
wt_614.pdb	A	4.24	0.12	0%	19	42	42

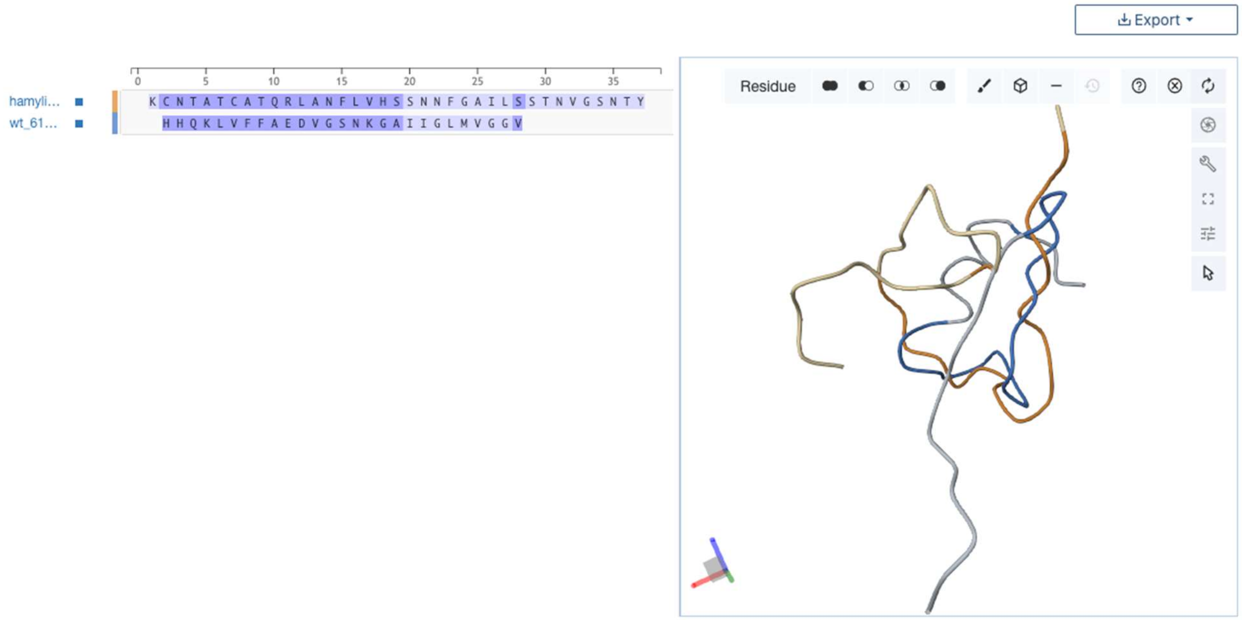

Entry	Chain	RMSD	TM-score	Identity	Equivalent Residues	Sequence Length	Modelled Residues
hamylin_1439.pdb	A	-	-	-	-	37	37
wt_614.pdb	A	1.56	0.14	0%	8	42	10

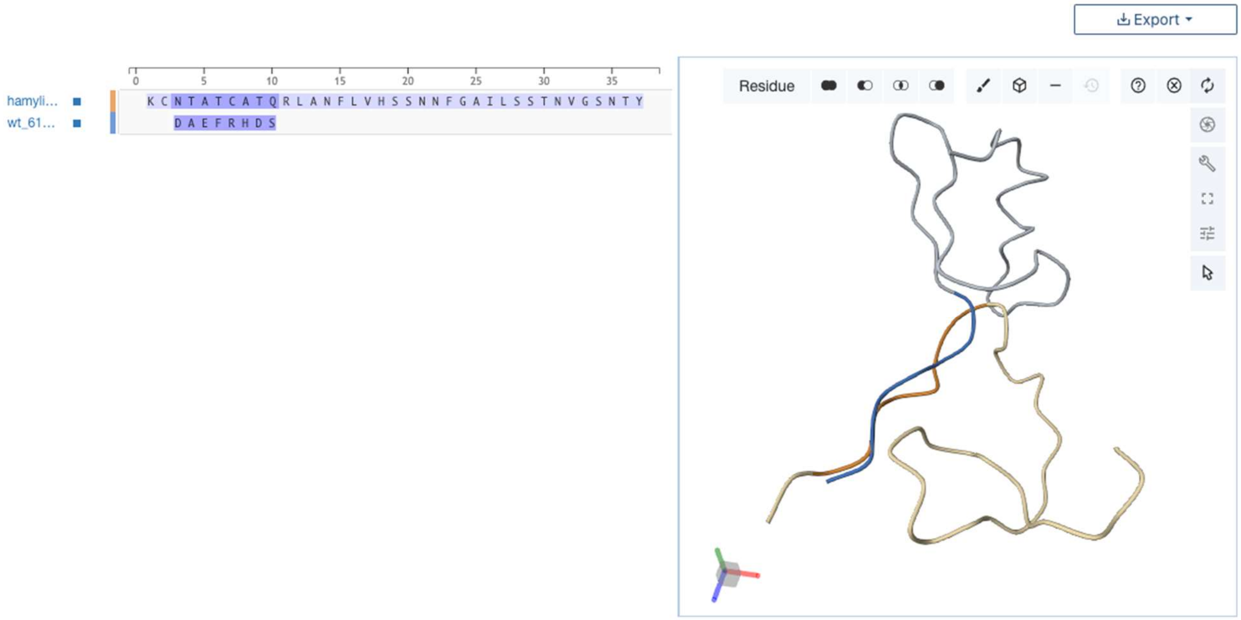

Hamylin_1439 – wt_327

Entry	Chain	RMSD	TM-score	Identity	Equivalent Residues	Sequence Length	Modelled Residues
hamylin_1439.pdb	A	-	-	-	-	37	37
wt_327.pdb	A	3.48	0.21	8%	23	42	42

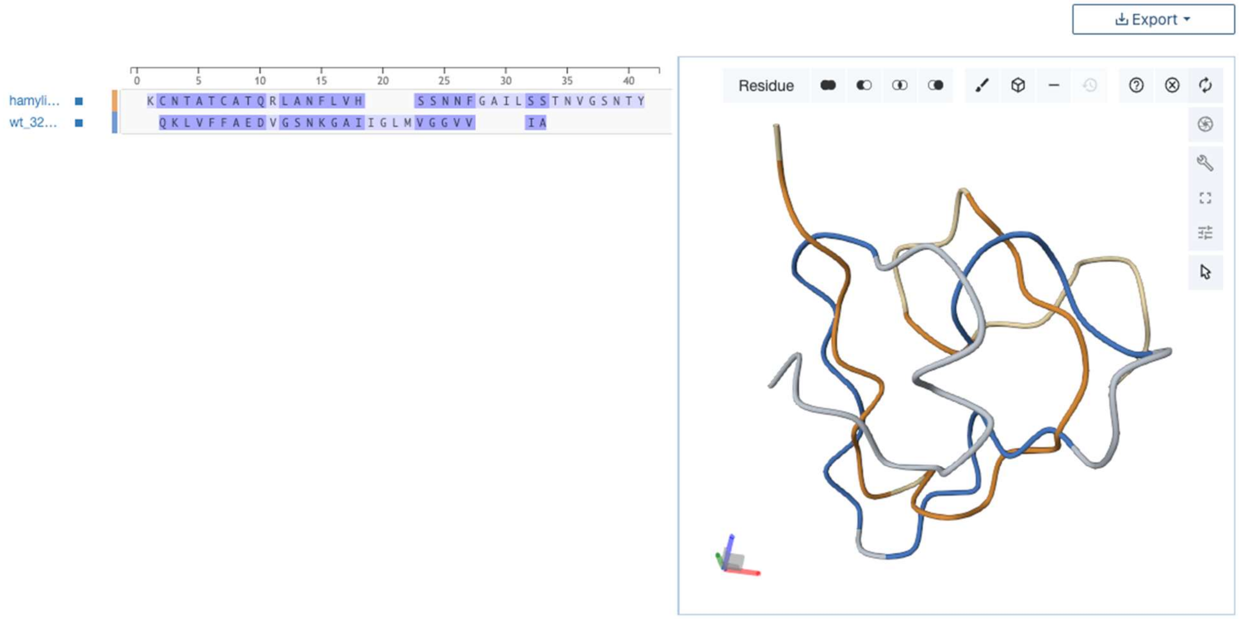

Entry	Chain	RMSD	TM-score	Identity	Equivalent Residues	Sequence Length	Modelled Residues
hamylin_1439.pdb	A	-	-	-	-	37	37
wt_327.pdb	A	1.7	0.13	10%	9	42	10

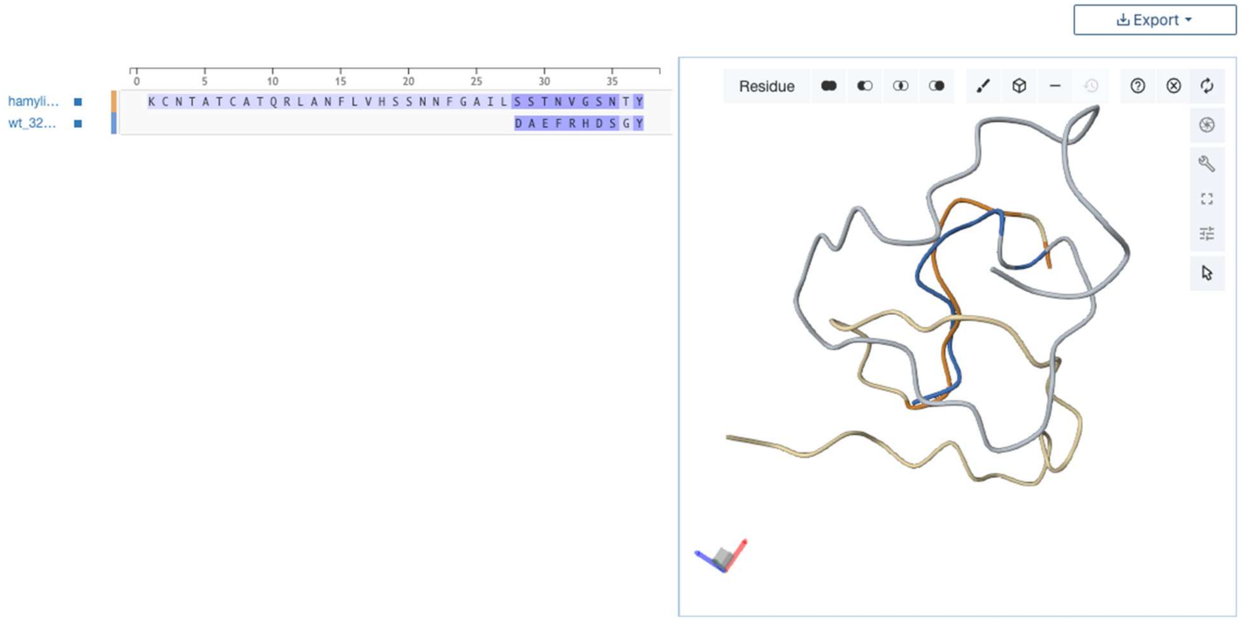

Hamylin_1439 – wt_89

Entry	Chain	RMSD	TM-score	Identity	Equivalent Residues	Sequence Length	Modelled Residues
hamylin_1439.pdb	A	-	-	-	-	37	37
wt_89.pdb	A	2.18	0.15	0%	11	42	42

Export

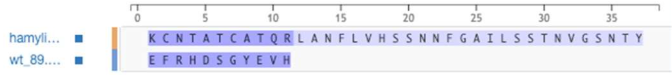

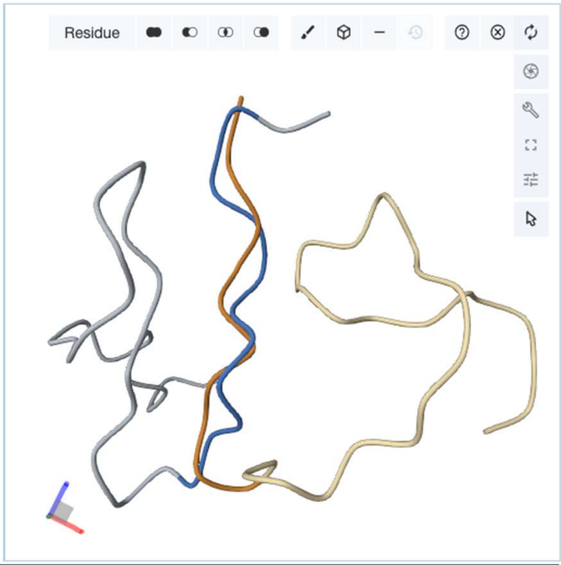

Entry	Chain	RMSD	TM-score	Identity	Equivalent Residues	Sequence Length	Modelled Residues
hamylin_1439.pdb	A	-	-	-	-	37	37
wt_89.pdb	A	1.55	0.14	11%	9	42	10

Export

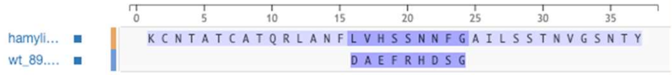

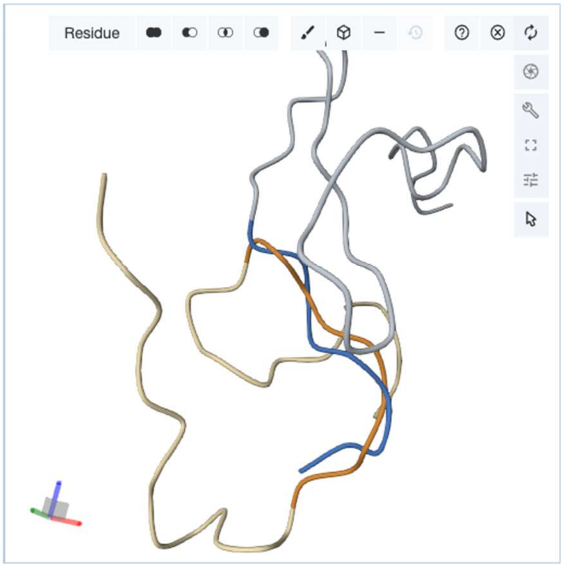

Hamylin_1336 – wt_1831

Entry	Chain	RMSD	TM-score	Identity	Equivalent Residues	Sequence Length	Modelled Residues
hamylin_1336.pdb	A	-	-	-	-	37	37
wt_1831.pdb	A	3.84	0.13	16%	16	42	42

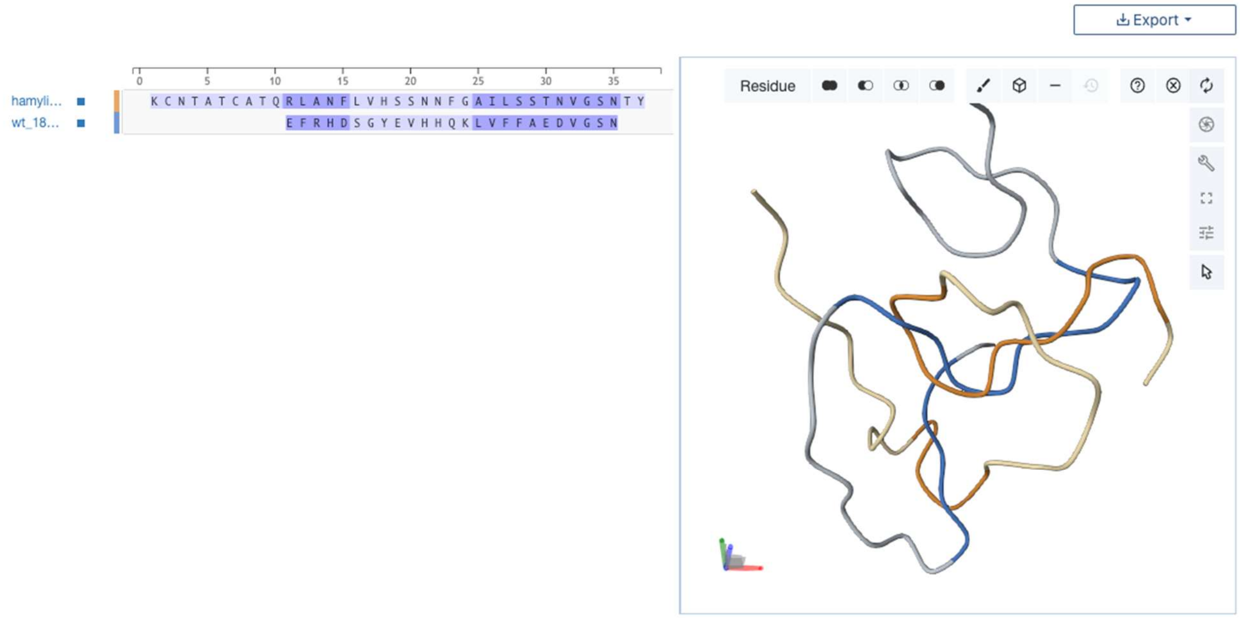

Entry	Chain	RMSD	TM-score	Identity	Equivalent Residues	Sequence Length	Modelled Residues
hamylin_1336.pdb	A	-	-	-	-	37	37
wt_1831.pdb	A	1.83	0.14	10%	10	42	10

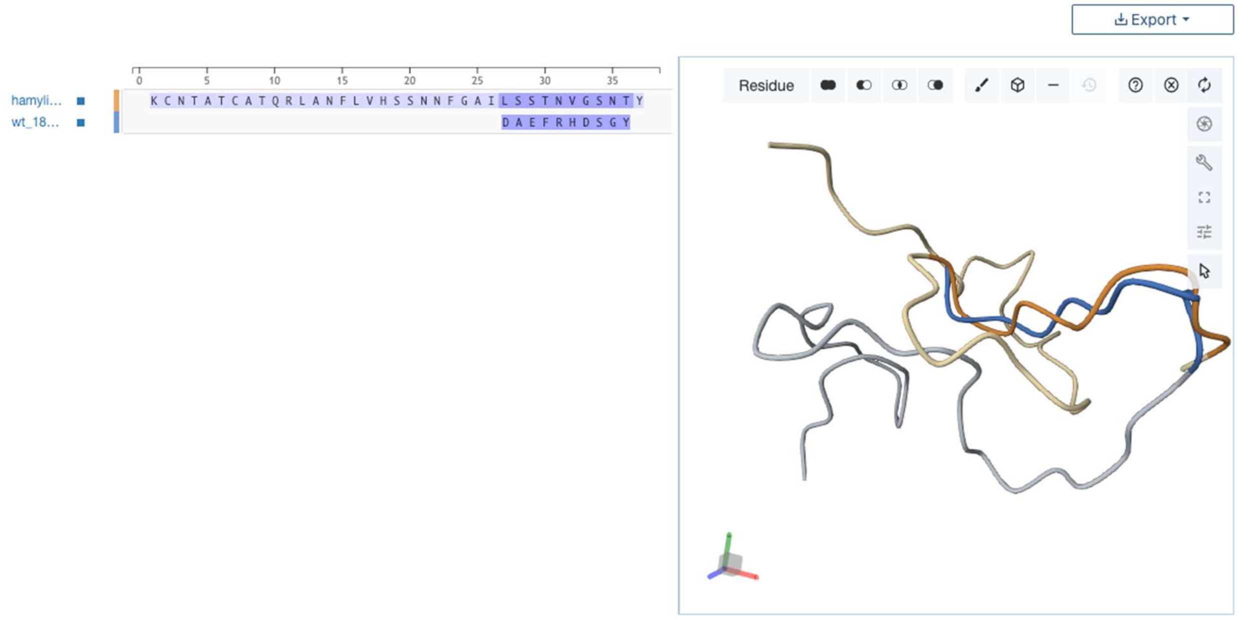

Hamylin_1336 – wt_1334

Entry	Chain	RMSD	TM-score	Identity	Equivalent Residues	Sequence Length	Modelled Residues
hamylin_1336.pdb	A	-	-	-	-	37	37
wt_1334.pdb	A	3.32	0.18	0%	19	42	42

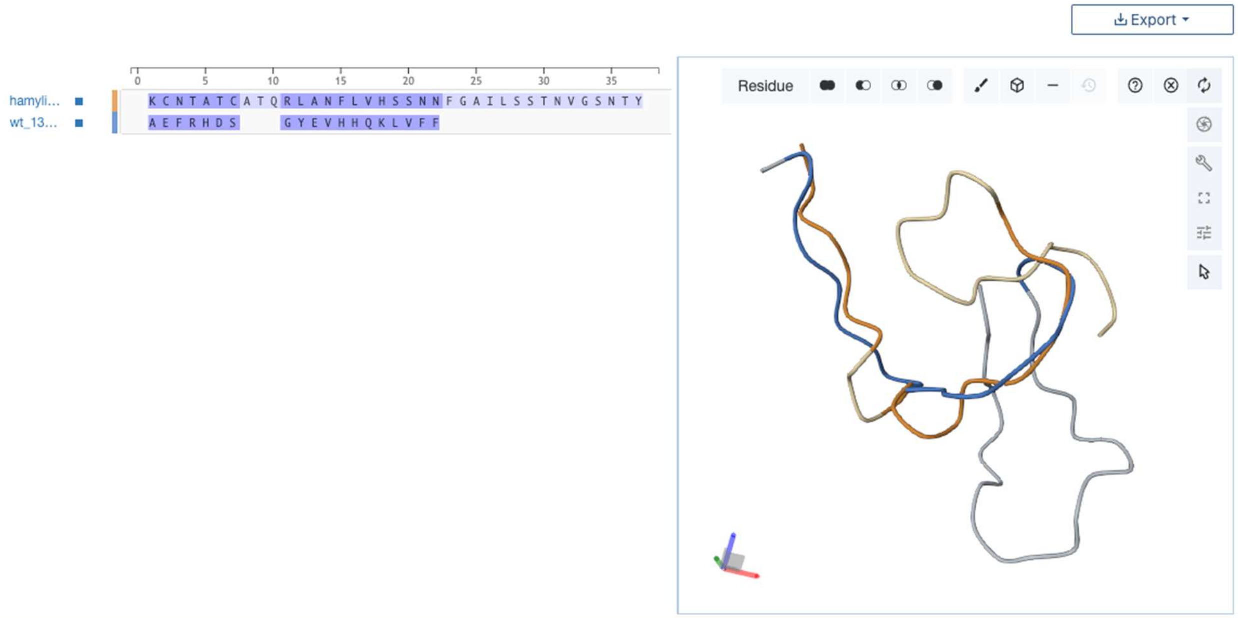

Entry	Chain	RMSD	TM-score	Identity	Equivalent Residues	Sequence Length	Modelled Residues
hamylin_1336.pdb	A	-	-	-	-	37	37
wt_1334.pdb	A	1.49	0.13	13%	8	42	10

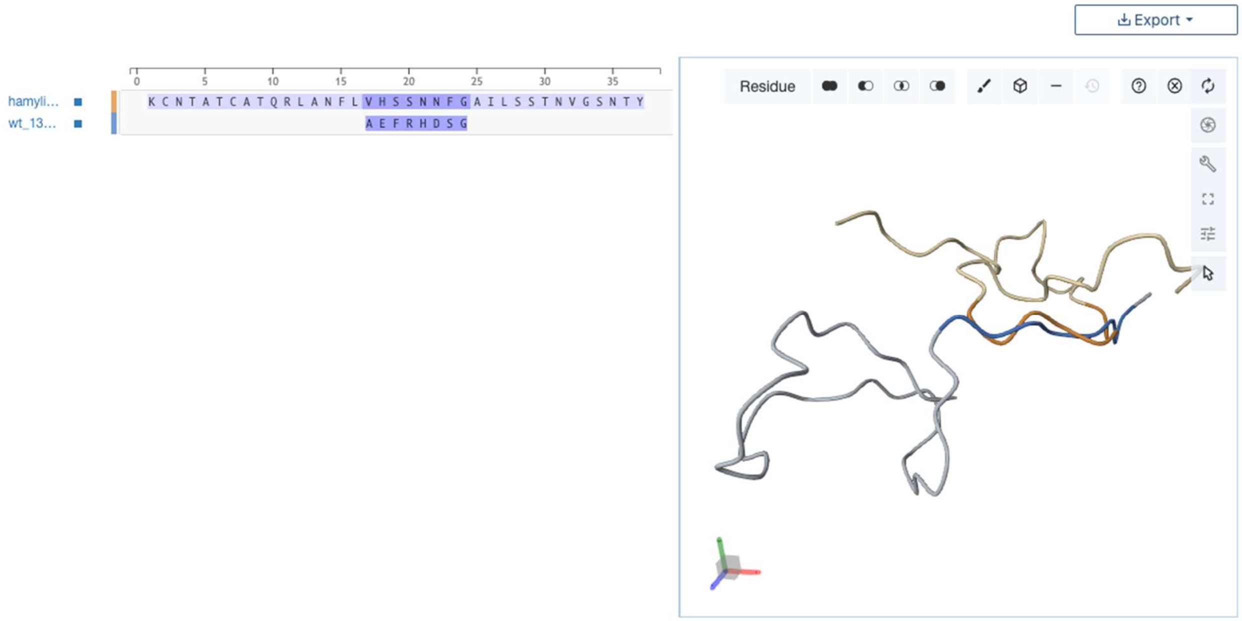

Hamylin_1336 -wt_1159

Entry	Chain	RMSD	TM-score	Identity	Equivalent Residues	Sequence Length	Modelled Residues
hamylin_1336.pdb	A	-	-	-	-	37	37
wt_1159.pdb	A	3.21	0.19	0%	20	42	42

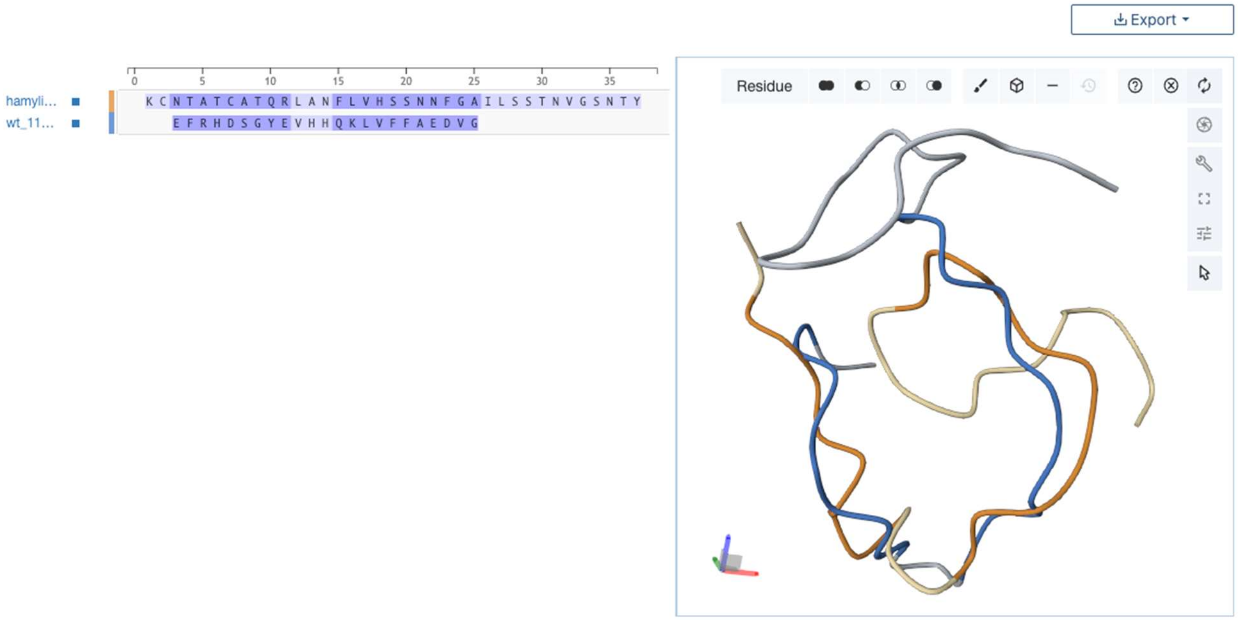

Entry	Chain	RMSD	TM-score	Identity	Equivalent Residues	Sequence Length	Modelled Residues
hamylin_1336.pdb	A	-	-	-	-	37	37
wt_1159.pdb	A	3.16	0.11	0%	10	42	10

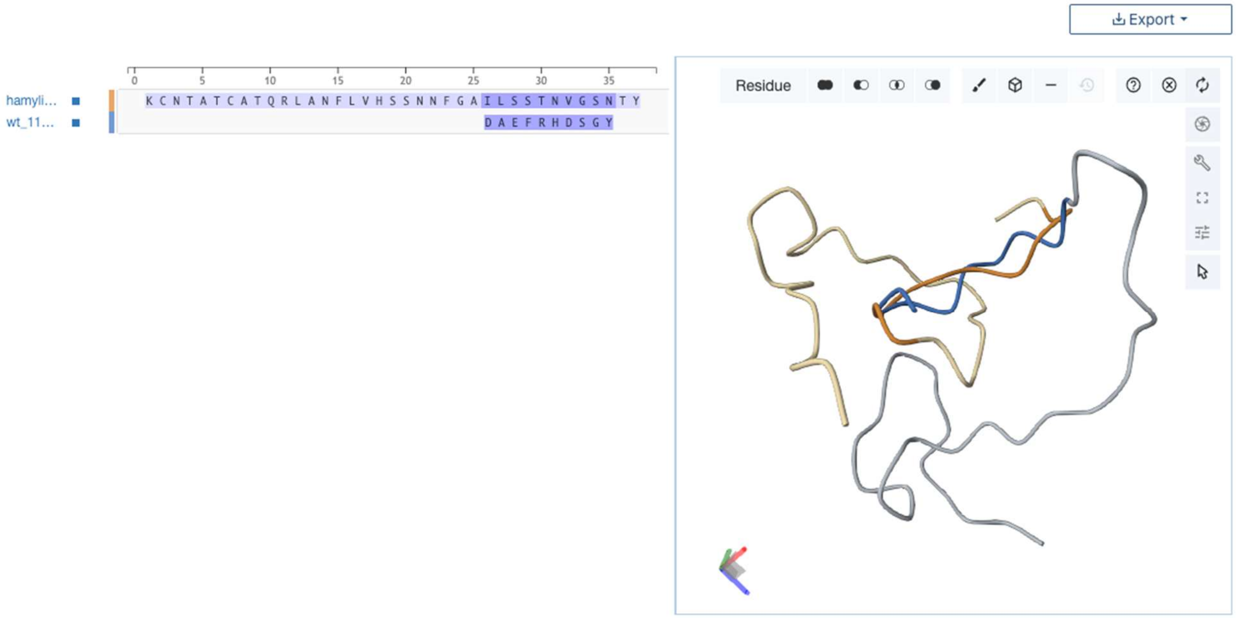

Hamylin_1336 – wt_749

Entry	Chain	RMSD	TM-score	Identity	Equivalent Residues	Sequence Length	Modelled Residues
hamylin_1336.pdb	A	-	-	-	-	37	37
wt_749.pdb	A	3.06	0.12	8%	13	42	42

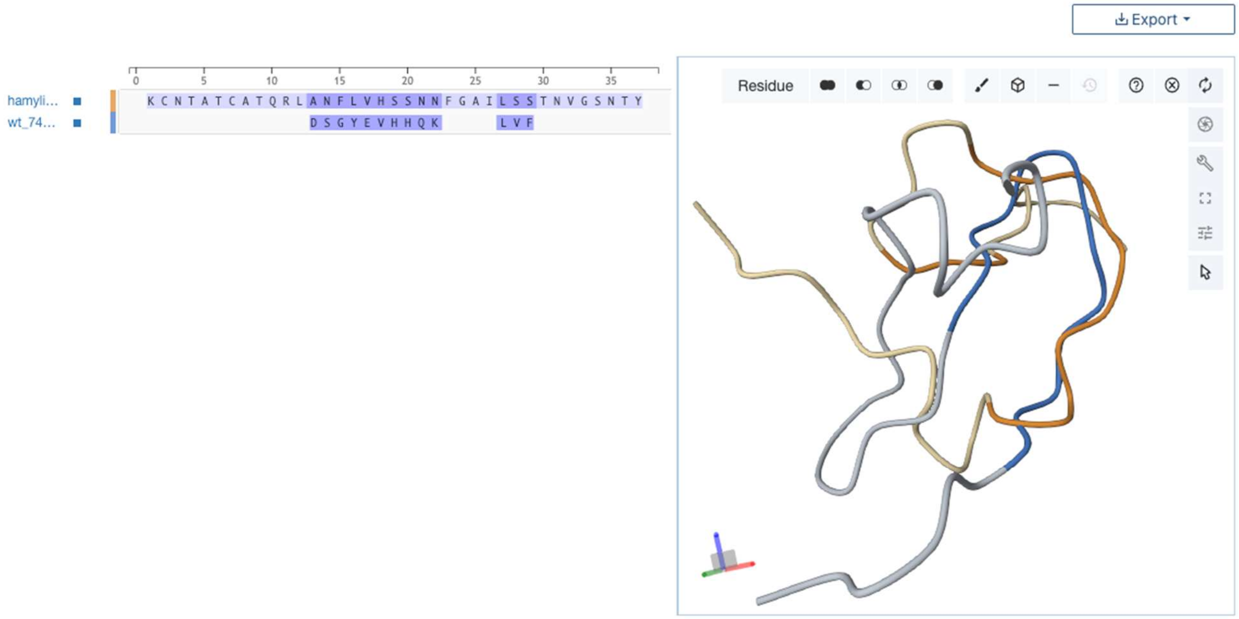

Entry	Chain	RMSD	TM-score	Identity	Equivalent Residues	Sequence Length	Modelled Residues
hamylin_1336.pdb	A	-	-	-	-	37	37
wt_749.pdb	A	2.26	0.13	0%	10	42	10

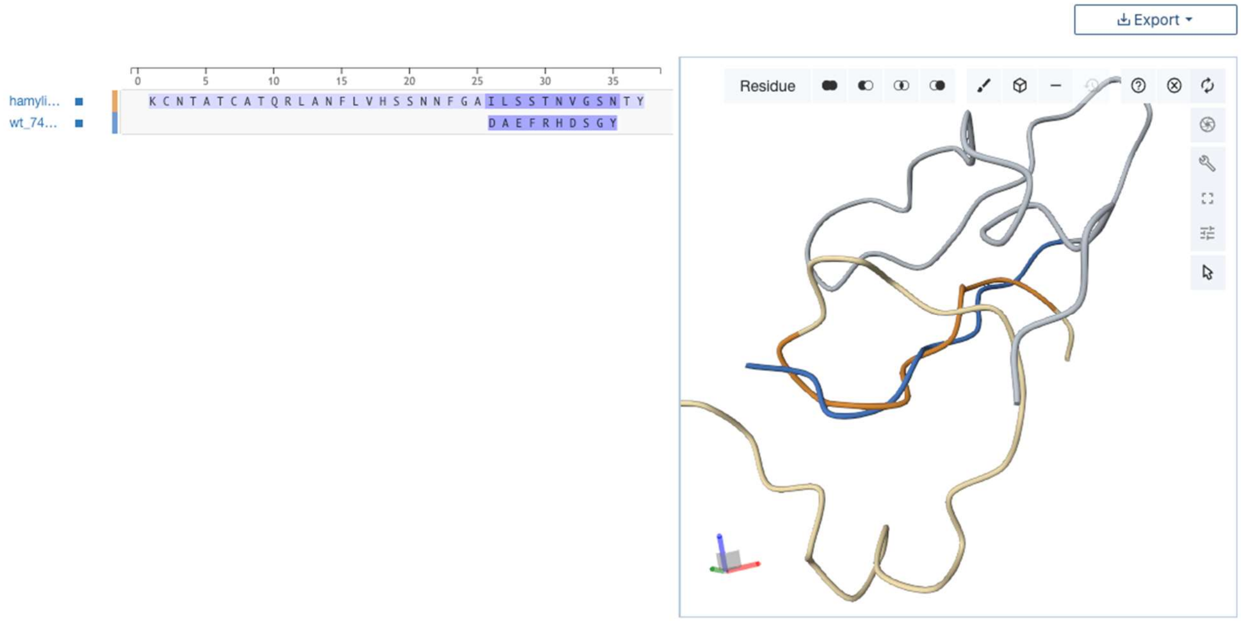

Hamylin_1336 – wt_614

Entry	Chain	RMSD	TM-score	Identity	Equivalent Residues	Sequence Length	Modelled Residues
hamylin_1336.pdb	A	-	-	-	-	37	37
wt_614.pdb	A	3.83	0.15	0%	19	42	42

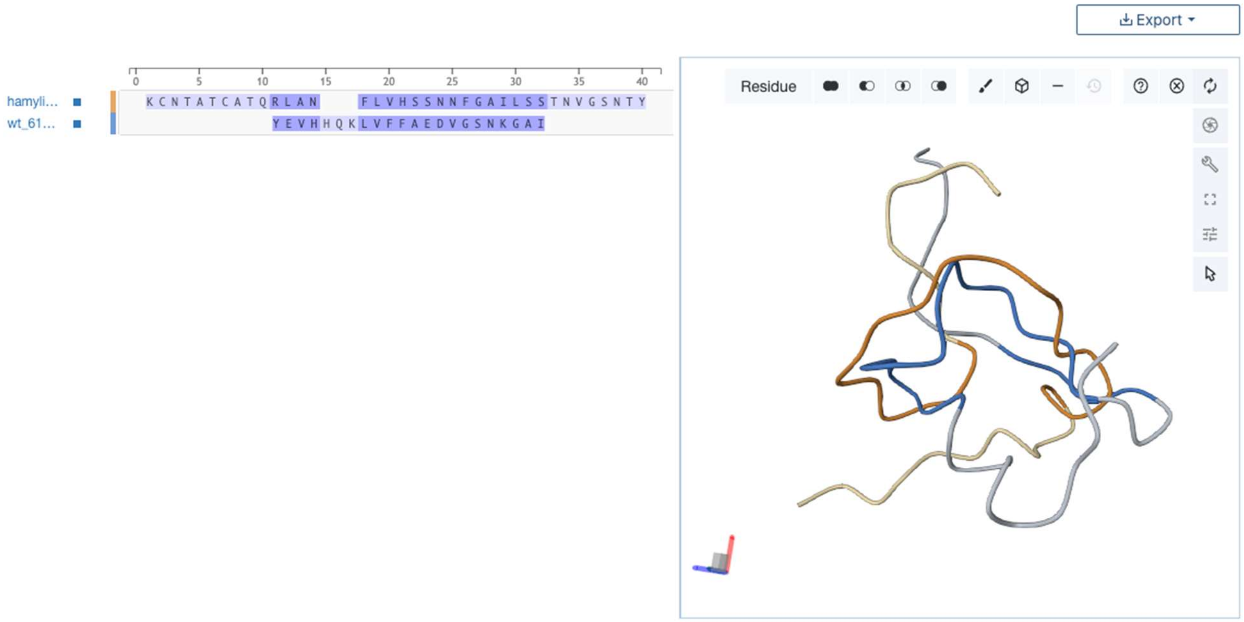

Entry	Chain	RMSD	TM-score	Identity	Equivalent Residues	Sequence Length	Modelled Residues
hamylin_1336.pdb	A	-	-	-	-	37	37
wt_614.pdb	A	1.35	0.14	0%	8	42	10

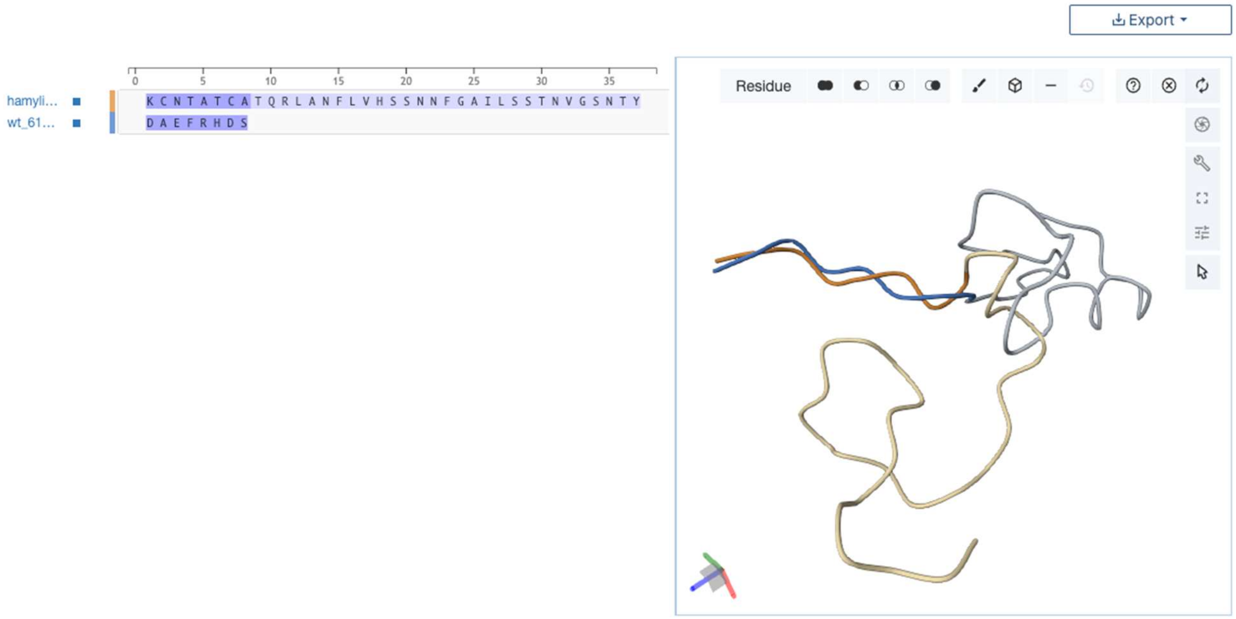

Hamylin_1336 – wt_327

Entry	Chain	RMSD	TM-score	Identity	Equivalent Residues	Sequence Length	Modelled Residues
hamylin_1336.pdb	A	-	-	-	-	37	37
wt_327.pdb	A	2.26	0.2	0%	16	42	42

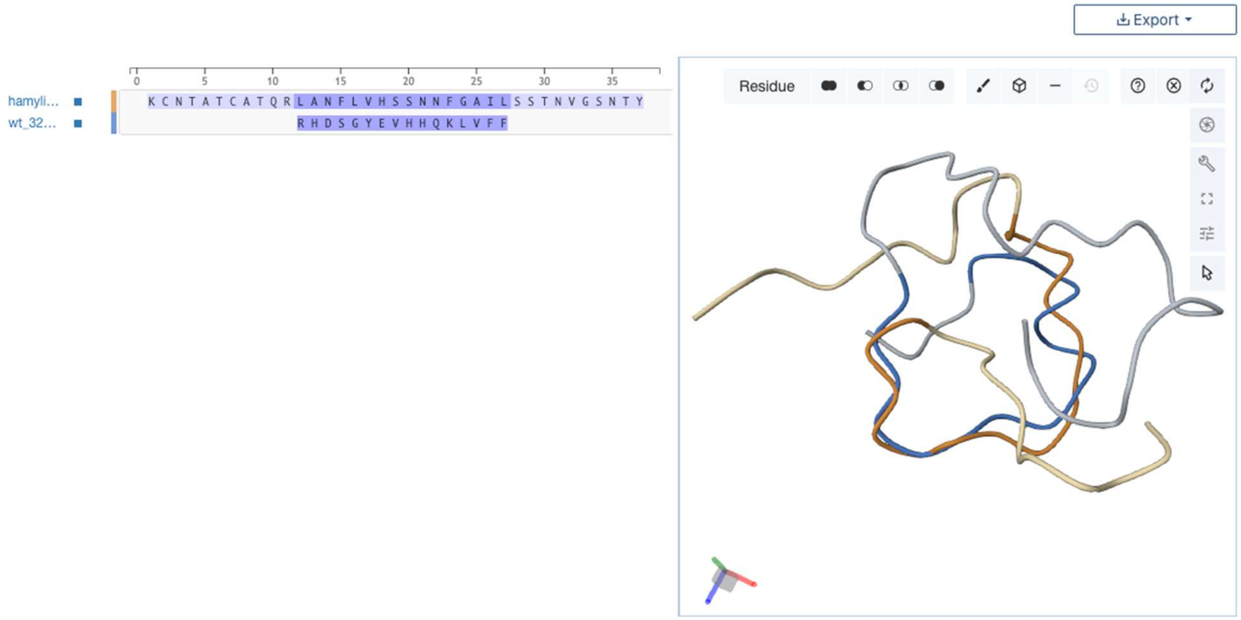

Entry	Chain	RMSD	TM-score	Identity	Equivalent Residues	Sequence Length	Modelled Residues
hamylin_1336.pdb	A	-	-	-	-	37	37
wt_327.pdb	A	2.28	0.12	10%	10	42	10

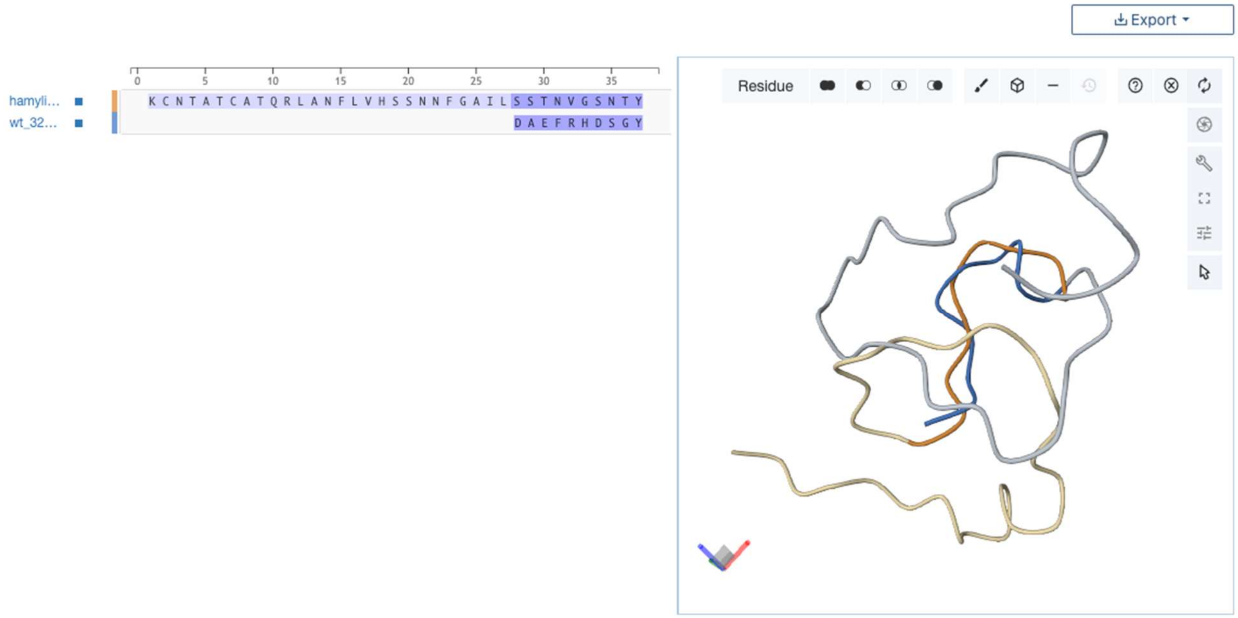

Hamylin_1336 – wt_89

Entry	Chain	RMSD	TM-score	Identity	Equivalent Residues	Sequence Length	Modelled Residues
hamylin_1336.pdb	A	-	-	-	-	37	37
wt_89.pdb	A	3.46	0.12	8%	13	42	42

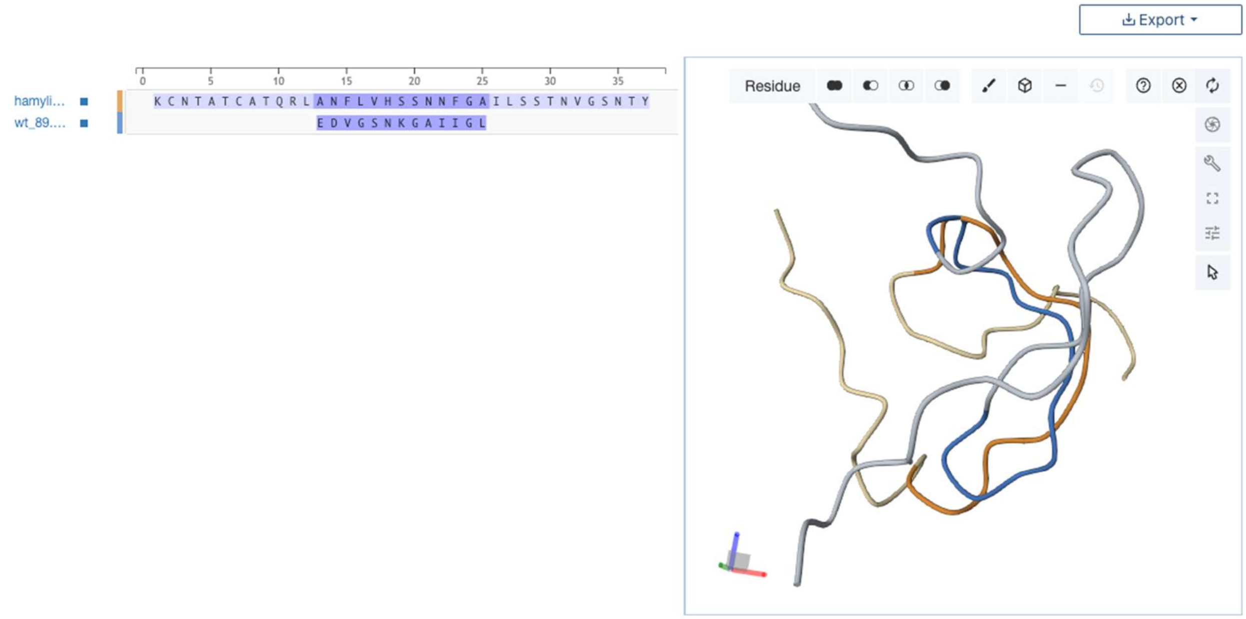

Entry	Chain	RMSD	TM-score	Identity	Equivalent Residues	Sequence Length	Modelled Residues
hamylin_1336.pdb	A	-	-	-	-	37	37
wt_89.pdb	A	1.74	0.13	11%	9	42	10

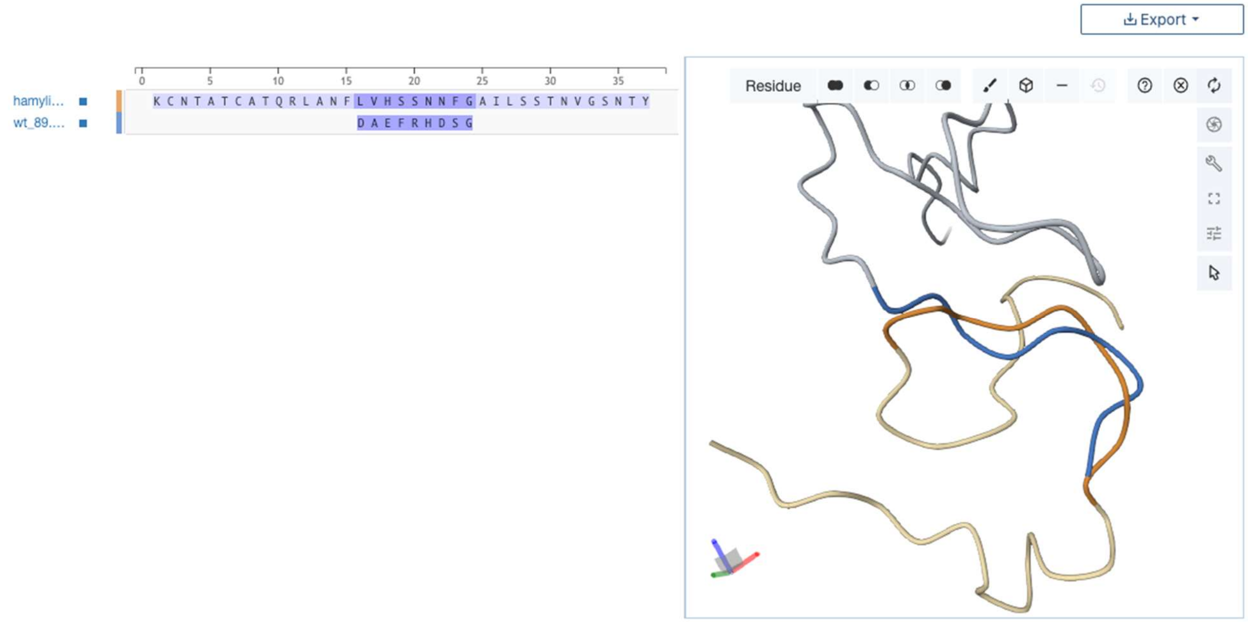

Hamylin_244 – wt_1831

Entry	Chain	RMSD	TM-score	Identity	Equivalent Residues	Sequence Length	Modelled Residues
hamylin_244.pdb	A	-	-	-	-	37	37
wt_1831.pdb	A	4.52	0.12	10%	18	42	42

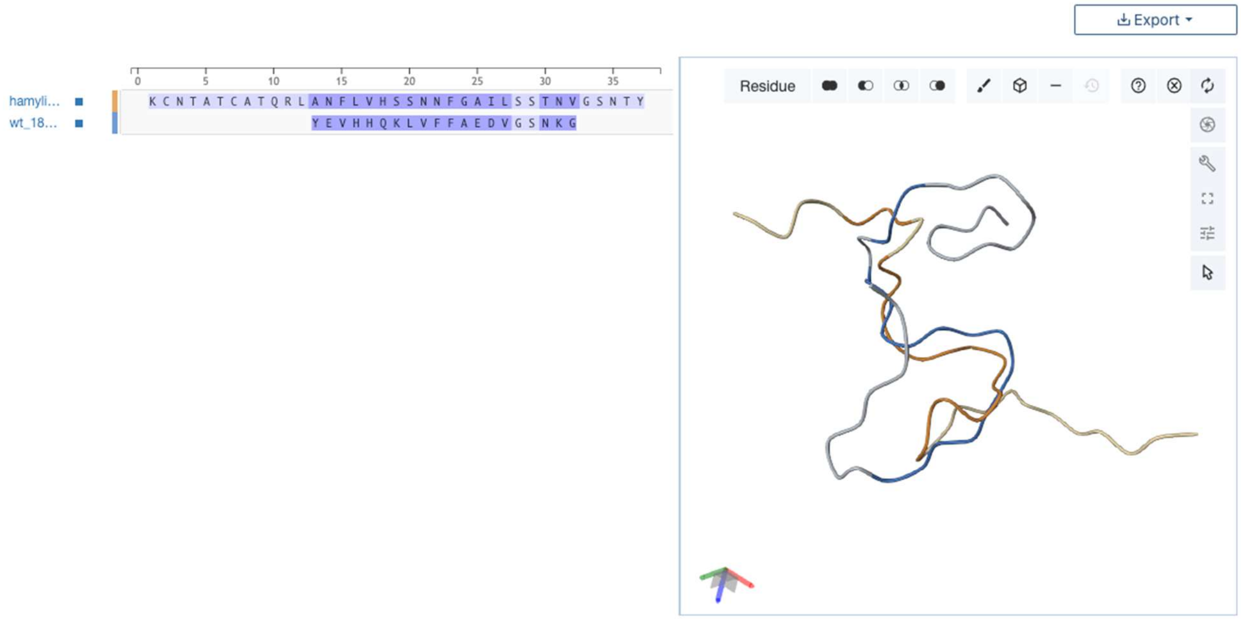

Entry	Chain	RMSD	TM-score	Identity	Equivalent Residues	Sequence Length	Modelled Residues
hamylin_244.pdb	A	-	-	-	-	37	37
wt_1831.pdb	A	3.16	0.1	0%	10	42	10

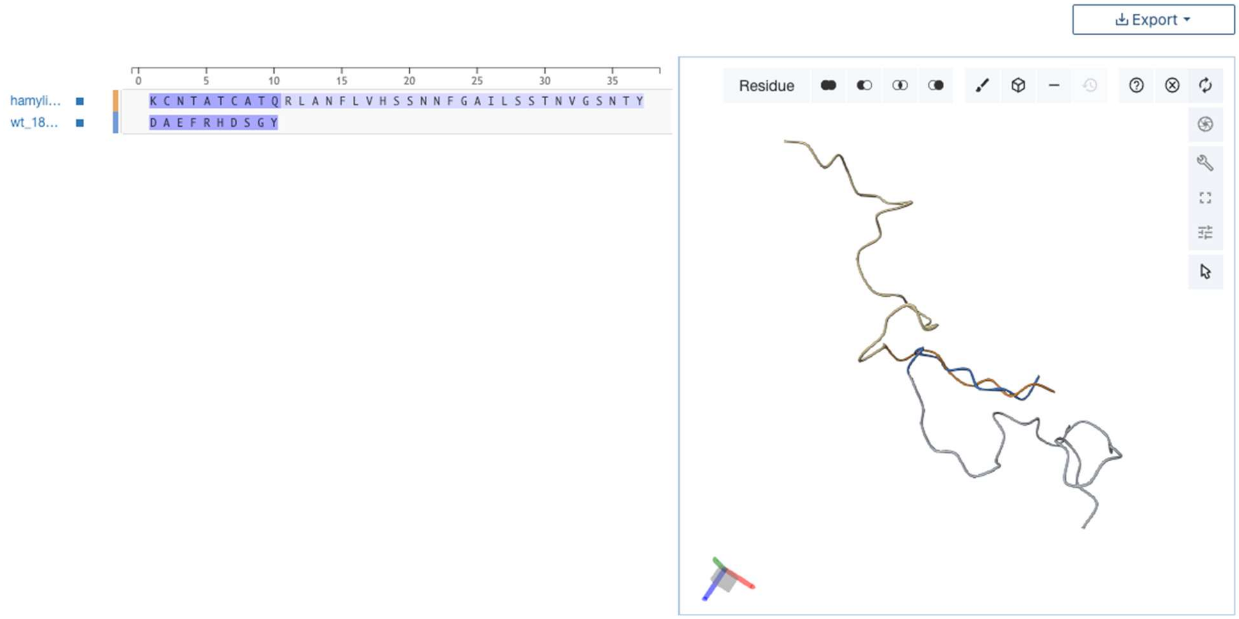

Hamylin_244 – wt_1334

Entry	Chain	RMSD	TM-score	Identity	Equivalent Residues	Sequence Length	Modelled Residues
hamylin_244.pdb	A	-	-	-	-	37	37
wt_1334.pdb	A	3.17	0.13	7%	15	42	42

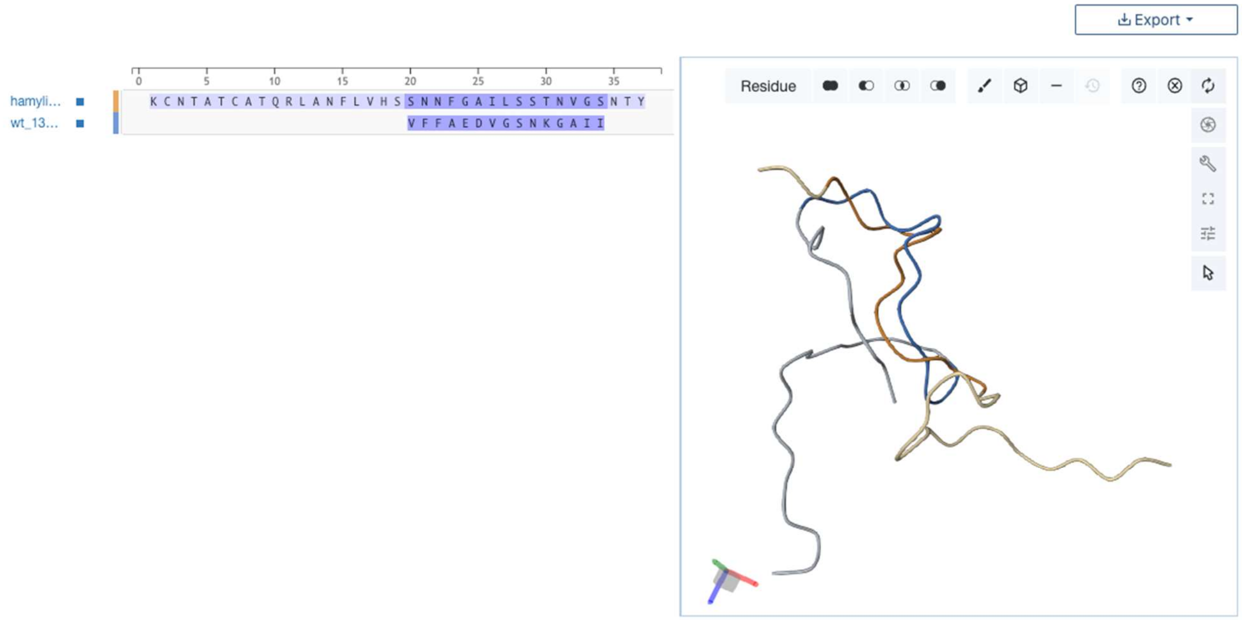

Entry	Chain	RMSD	TM-score	Identity	Equivalent Residues	Sequence Length	Modelled Residues
hamylin_244.pdb	A	-	-	-	-	37	37
wt_1334.pdb	A	1.66	0.12	0%	8	42	10

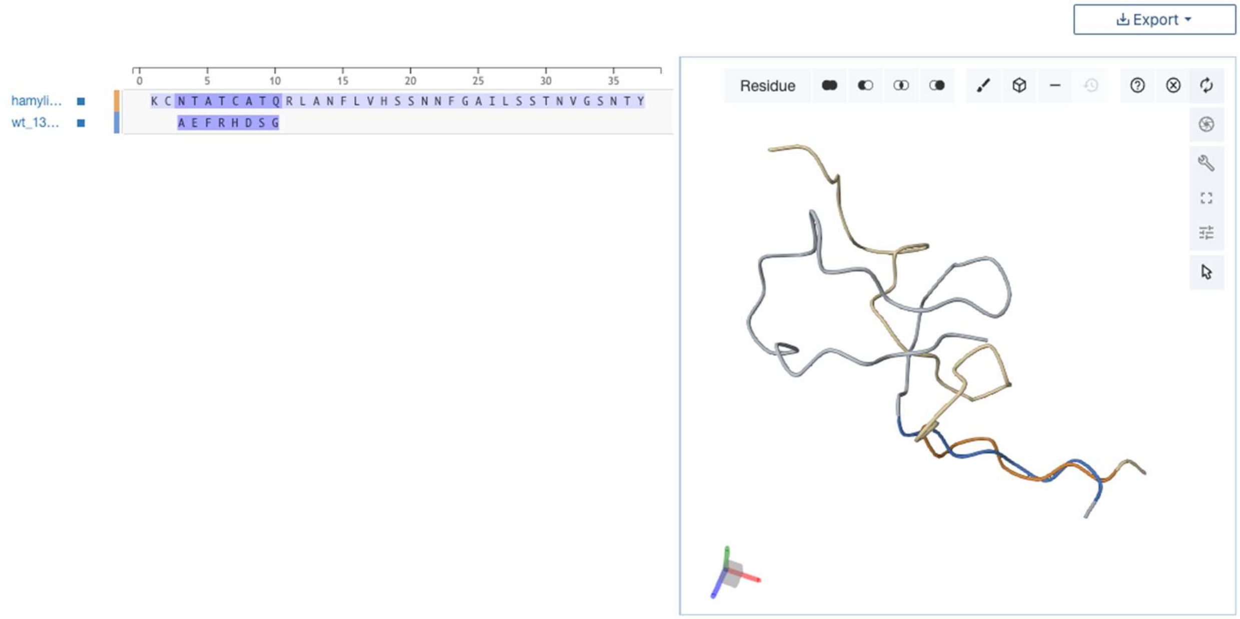

Hamylin_244 – wt_1159

Entry	Chain	RMSD	TM-score	Identity	Equivalent Residues	Sequence Length	Modelled Residues
hamylin_244.pdb	A	-	-	-	-	37	37
wt_1159.pdb	A	3.06	0.15	10%	14	42	42

Export

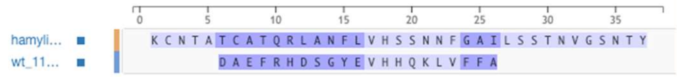

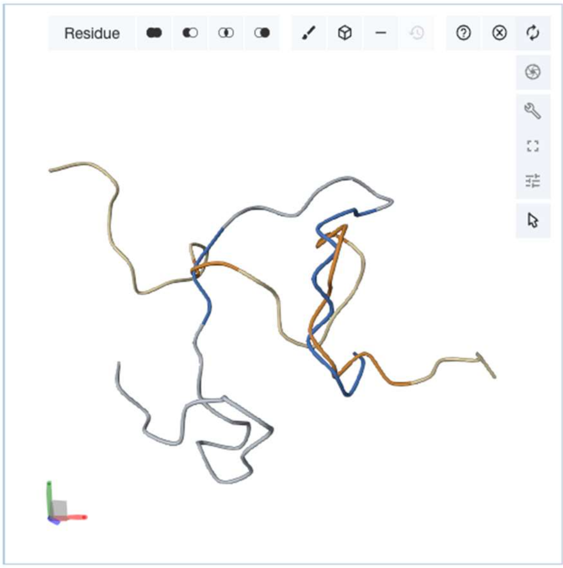

Entry	Chain	RMSD	TM-score	Identity	Equivalent Residues	Sequence Length	Modelled Residues
hamylin_244.pdb	A	-	-	-	-	37	37
wt_1159.pdb	A	0.95	0.17	0%	8	42	10

Export

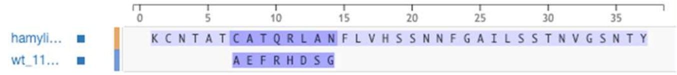

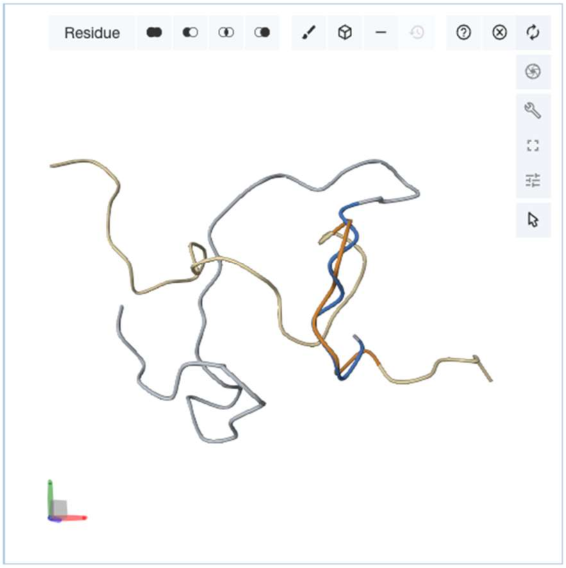

Hamylin_244 – wt_749

Entry	Chain	RMSD	TM-score	Identity	Equivalent Residues	Sequence Length	Modelled Residues
hamylin_244.pdb	A	-	-	-	-	37	37
wt_749.pdb	A	0.8	0.16	13%	8	42	42

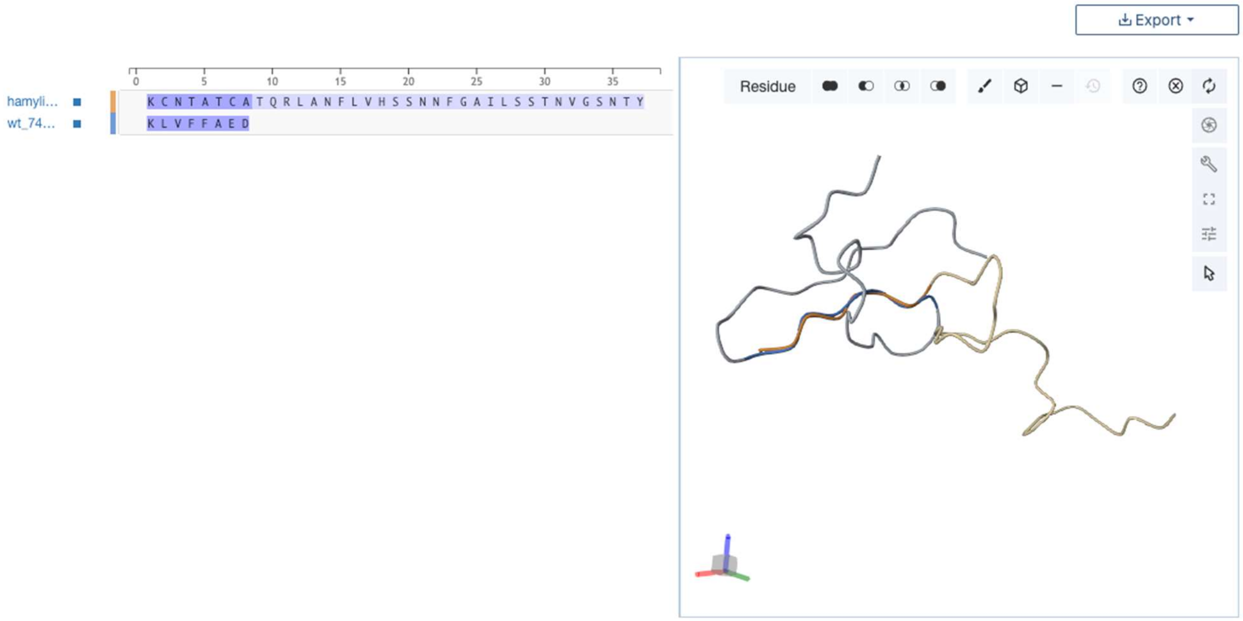

Entry	Chain	RMSD	TM-score	Identity	Equivalent Residues	Sequence Length	Modelled Residues
hamylin_244.pdb	A	-	-	-	-	37	37
wt_749.pdb	A	1.39	0.13	0%	8	42	10

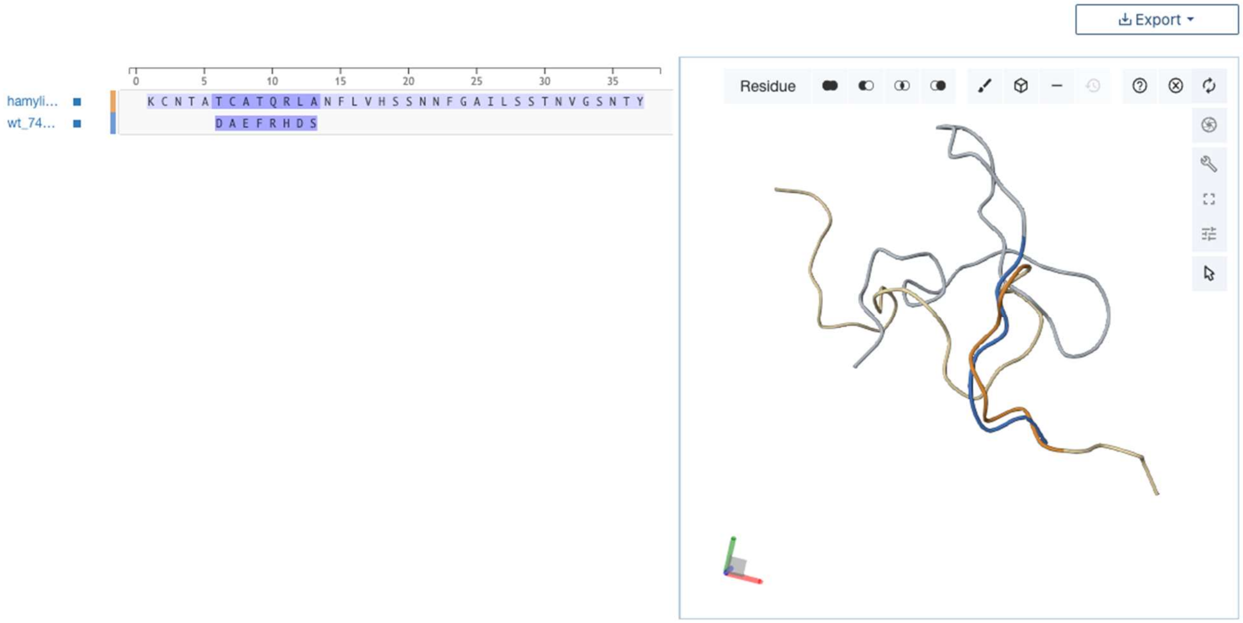

Hamylin_244 – wt_614

Entry	Chain	RMSD	TM-score	Identity	Equivalent Residues	Sequence Length	Modelled Residues
hamylin_244.pdb	A	-	-	-	-	37	37
wt_614.pdb	A	4.92	0.13	0%	22	42	42

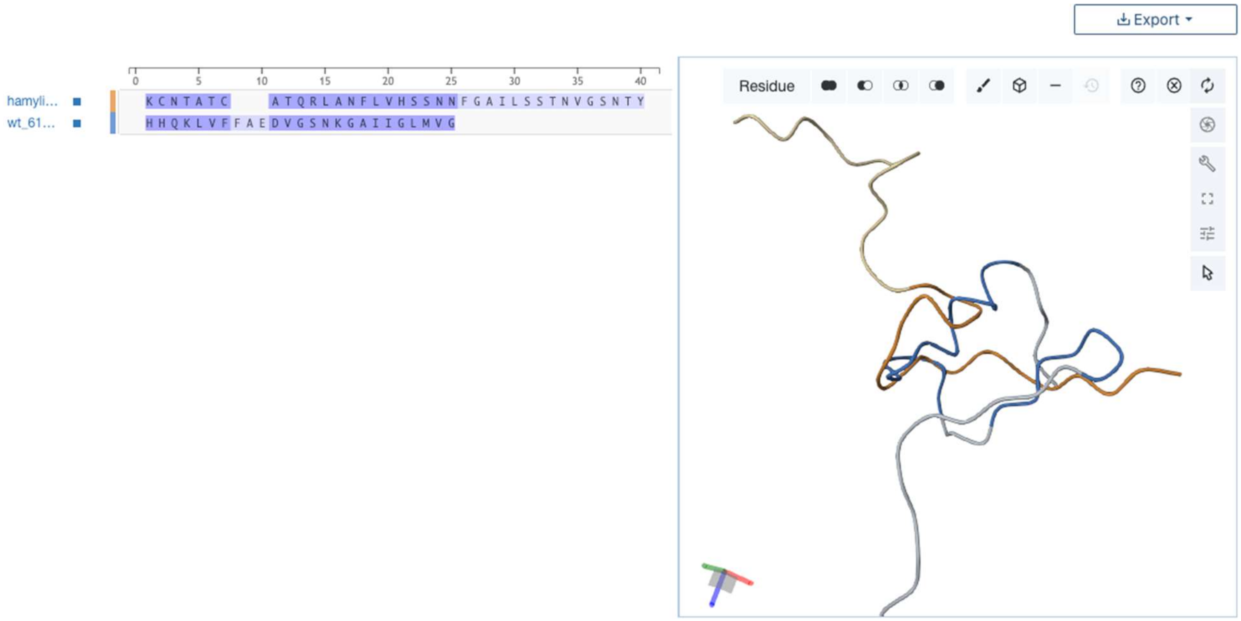

Entry	Chain	RMSD	TM-score	Identity	Equivalent Residues	Sequence Length	Modelled Residues
hamylin_244.pdb	A	-	-	-	-	37	37
wt_614.pdb	A	1.47	0.14	0%	8	42	10

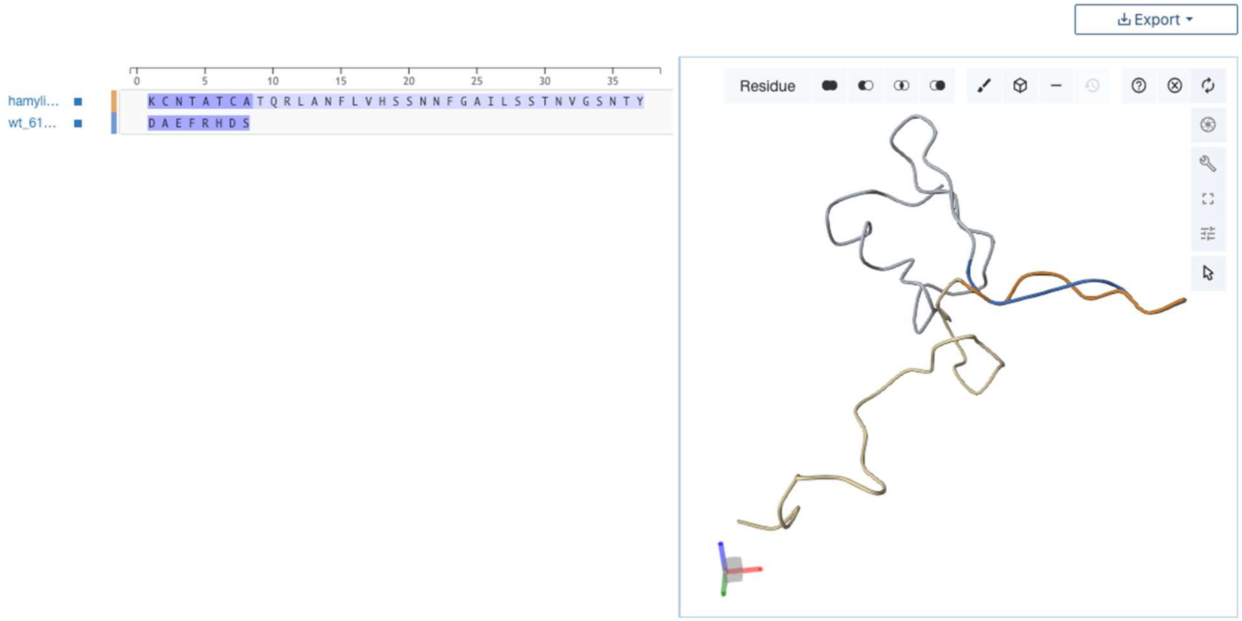

Hamylin_244 – wt_327

Entry	Chain	RMSD	TM-score	Identity	Equivalent Residues	Sequence Length	Modelled Residues
hamylin_244.pdb	A	-	-	-	-	37	37
wt_327.pdb	A	0.75	0.17	0%	8	42	42

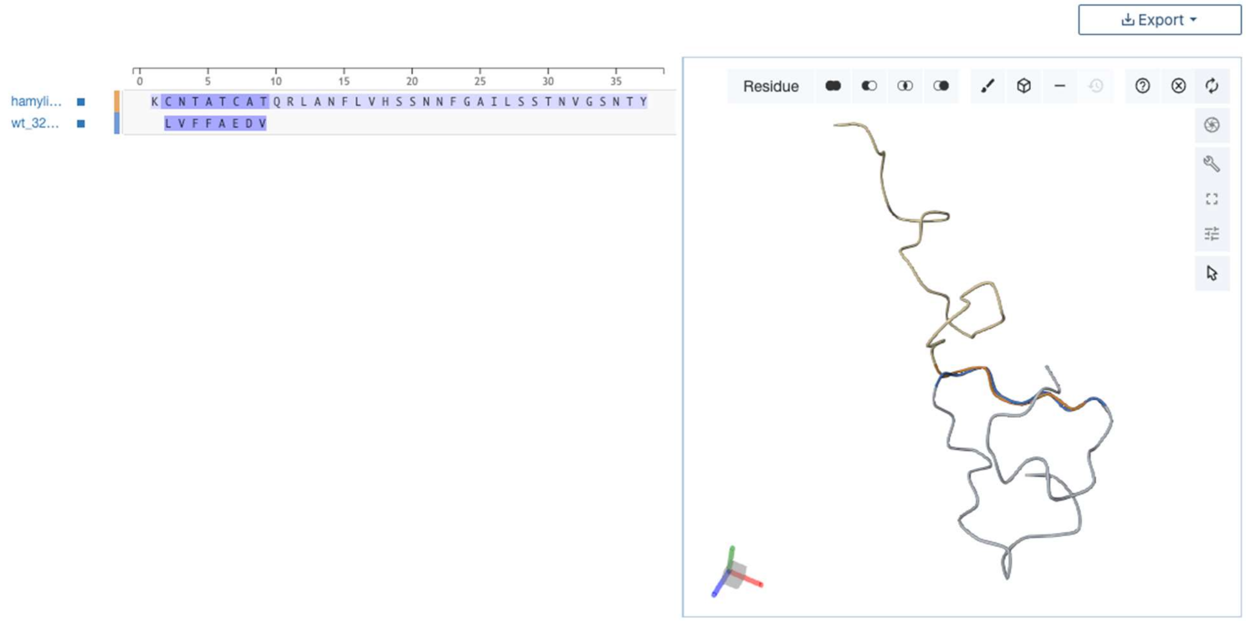

Entry	Chain	RMSD	TM-score	Identity	Equivalent Residues	Sequence Length	Modelled Residues
hamylin_244.pdb	A	-	-	-	-	37	37
wt_327.pdb	A	1.28	0.14	0%	8	42	10

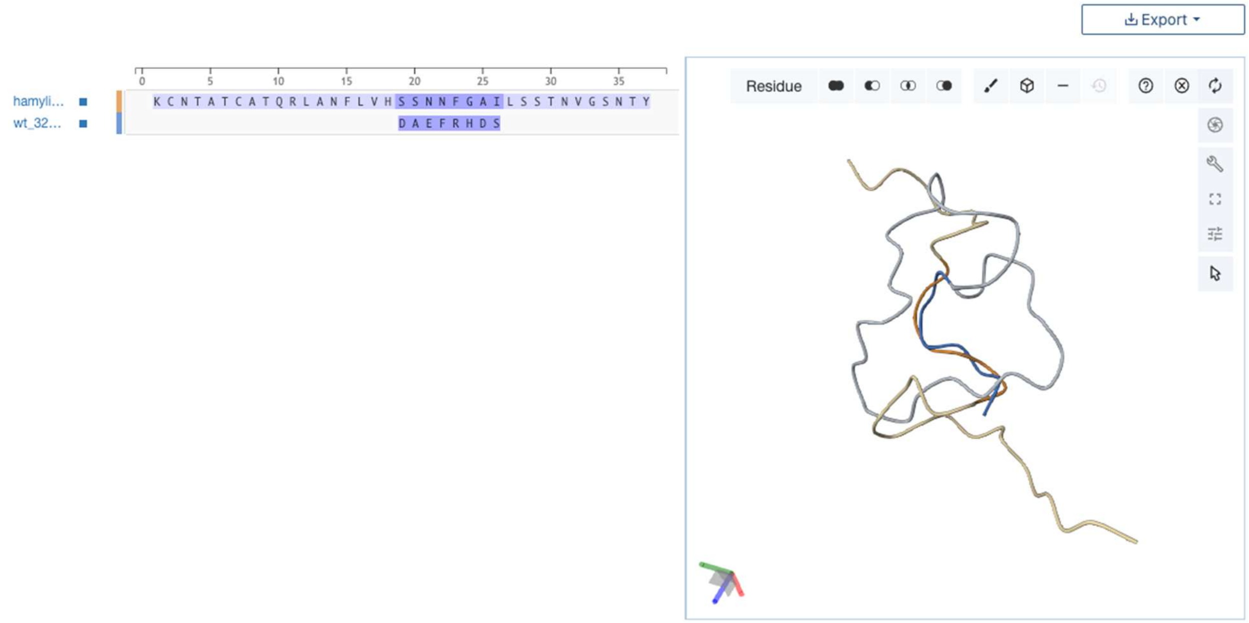

Hamylin_244 – wt_89

Entry	Chain	RMSD	TM-score	Identity	Equivalent Residues	Sequence Length	Modelled Residues
hamylin_244.pdb	A	-	-	-	-	37	37
wt_89.pdb	A	1.6	0.16	0%	10	42	42

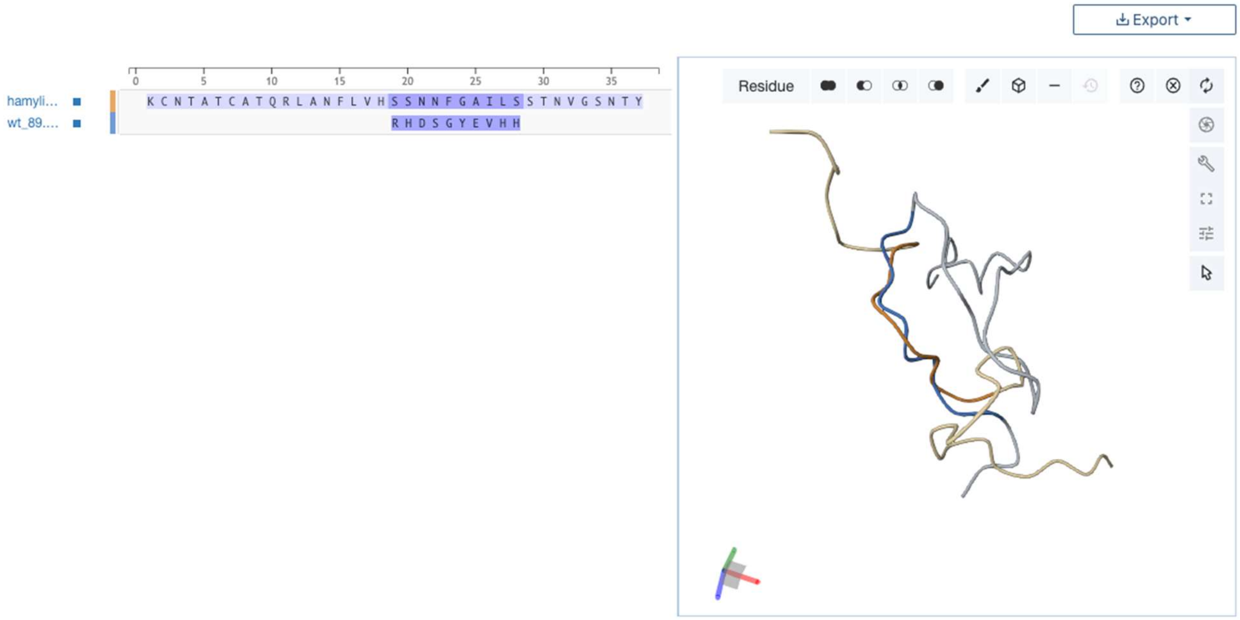

Entry	Chain	RMSD	TM-score	Identity	Equivalent Residues	Sequence Length	Modelled Residues
hamylin_244.pdb	A	-	-	-	-	37	37
wt_89.pdb	A	1.19	0.16	0%	8	42	10

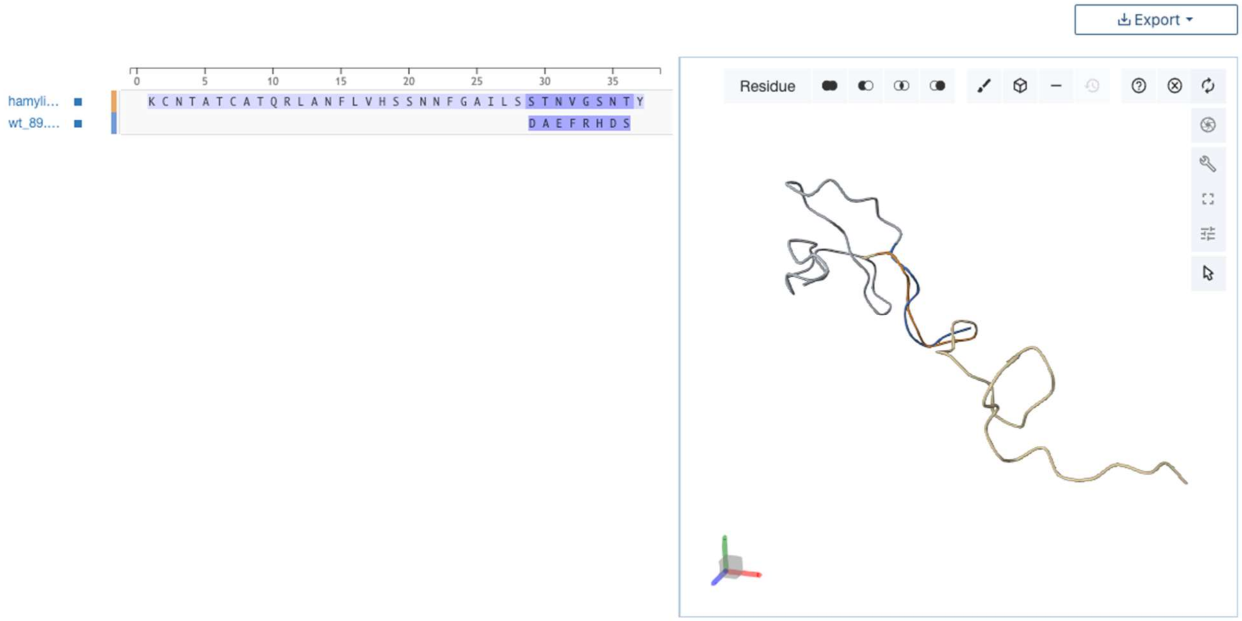

Supplement: Supplementary file 1 [file biomolecules-15-00089-s001.zip › biomolecules-3264739-supplementary File S1.pdf]
